# Roadmap on biomaterials for women’s health

**DOI:** 10.1088/2515-7639/ac90ee

**Published:** 2022-12-22

**Authors:** Kaitlin Fogg, Ning-Hsuan Tseng, Shelly R Peyton, Pieper Holeman, Shannon Mc Loughlin, John P Fisher, Allison Sutton, Ariella Shikanov, Juan S Gnecco, Katrina M Knight, Emily M Slaby, Jessica D Weaver, Nicole N Hashemi, Yali Zhang, Michael D House, Brandon J Vogt, Brian A Aguado, John C Bradford, Jennifer L Robinson, Patricia K Thomas, Anthony G Lau, Michelle L Oyen

**Affiliations:** 1 School of Chemical, Biological and Environmental Engineering, Oregon State University, United States of America; 2 Molecular and Cellular Biology Graduate Program, University of Massachusetts Amherst, United States of America; 3 Department of Chemical Engineering, University of Massachusetts Amherst, MA, United States of America; 4 Fischell Department of Bioengineering, University of Maryland, College Park, MD, United States of America; 5 Department of Biomedical Engineering, University of Michigan, Ann Arbor, MI 48109, United States of America; 6 Program in Cellular and Molecular Biology, University of Michigan, Ann Arbor, MI 48109, United States of America; 7 Department of Biomedical Engineering, Tufts University, Medford, MA, United States of America; 8 Department of Bioengineering, University of Pittsburgh, Pittsburgh, PA, United States of America; 9 School of Biological and Health Systems Engineering, Arizona State University, AZ, United States of America; 10 Department of Mechanical Engineering, Iowa State University, Ames, IA, United States of America; 11 Mother Infant Research Institute, Tufts Medical Center, Boston, MA, United States of America; 12 Department of Obstetrics and Gynecology, Division of Maternal Fetal Medicine, Tufts Medical Center, MA, United States of America; 13 Department of Bioengineering, University of California San Diego, La Jolla, CA, United States of America; 14 Sanford Consortium for Regenerative Medicine, La Jolla, CA, United States of America; 15 Bioengineering Graduate Program, University of Kansas, Lawrence, KS, United States of America; 16 Department of Chemical and Petroleum Engineering, University of Kansas, Lawrence, KS, United States of America; 17 The College of New Jersey, Department of Biomedical Engineering, Ewing, NJ, United States of America; 18 Department of Biomedical Engineering, Wake Forest University School of Medicine, Winston-Salem, NC, United States of America; 19 Virginia Tech-Wake Forest University School of Biomedical Engineering and Sciences, NC, United States of America; 20 Center for Women’s Health Engineering and Department of Biomedical Engineering, Washington University in St. Louis, St. Louis, MO, United States of America; 21 Guest editors of the Roadmap.

**Keywords:** women’s health, biomaterials, breast cancer, reproductive organs, sex differences

## Abstract

The application of engineering tools and techniques to studying women’s health, including biomaterials-based approaches, is a research field experiencing robust growth. Biomaterials are natural or synthetic materials used to repair or replace damaged tissues or organs or replicate an organ’s physiological function. However, in addition to *in vivo* applications, there has been substantial recent interest in biomaterials for *in vitro* systems. Such artificial tissues and organs are employed in drug discovery, functional cell biological investigations, and basic research that would be ethically impossible to conduct in living women. This Roadmap is a collection of 11 sections written by leading and up-and-coming experts in this field who review and discuss four aspects of biomaterials for women’s health. These include conditions that disproportionately but not exclusively affect women (e.g. breast cancer), conditions unique to female reproductive organs, in both non-pregnant and pregnant states, and sex differences in non-reproductive tissues (e.g. the cardiovascular system). There is a strong need to develop this exciting field, with the potential to materially influence women’s lives worldwide.

## 1. Guest editor introduction

*Kaitlin Fogg*^1^ *and Michelle L Oyen*^2^

^1^ School of Chemical, Biological and Environmental Engineering, Oregon State University, United States of America

^2^ Center for Women’s Health Engineering and Department of Biomedical Engineering, Washington University in St. Louis, St. Louis, MO, United States of America

Women’s health has been a historically underserved area within medicine. Recent analysis demonstrated that inventions associated with women’s health are disproportionately designed by female inventors [1]. This reveals twin problems in the development pipeline, the lack of female engineers and inventors, and a lack of basic science knowledge in the field. Recognition is growing that there are unique aspects of women’s health that have been understudied in the medical research community, also likely related to the underrepresentation of women within the STEM fields. Recent analysis has shown that funding for research into women’s health lags that of diseases that are gender neutral or that solely or disproportionately affect men [2].

The field of Biomaterials—including sub-fields such as drug delivery, tissue engineering (TE), biomimetic model materials, and biofabrication techniques—has seen rapid growth in applications in women’s health in the last decade. Recent special journal issues [3–6] and review articles [7–11] have highlighted these growing applications and have all been published within the last 3 years. This demonstrates the exciting growth of a new emerging field of scientific and engineering endeavors with the potential to make dramatic basic science discoveries that translate to clinical practice in the service of improving women’s health. This Roadmap article covers a sample of overviews on how biomaterials are being used in Women’s Health research. The articles are grouped into four topics: breast cancer and breast reconstruction; non-pregnant reproductive organs; pregnancy; and sex differences in non-reproductive organs and systems.

### Breast cancer and breast reconstruction

The first section contains two articles on biomaterials related to the breast. Tseng and Payton review developments leading to biofidelic *in vitro* breast cancer models based on naturally derived and synthetic biomaterials. They discuss how these systems model the tumor microenvironment (TME) and consider the effects of matrix stiffness on cell behavior. They further consider recent advances in the use of microfluidic devices and three-dimensional (3D) bioprinting techniques. Next, Holeman, McLoughlin and Fisher consider biomaterials in reconstruction of the nipple-areolar complex (NAC) following total mastectomies. They note that long-term satisfaction rates following breast reconstruction surgery (BRS) are reasonably low, and they consider TE alternatives to existing approaches. Their section highlights the importance of emotional well-being in patients following the trauma of major cancer surgery. Biomaterials approaches thus have potential for saving lives through fundamental cancer research and for improving saved lives by improving body image and self-confidence of post-op patients.

### Reproductive organs—non-pregnant

The second section of this Roadmap is comprised of three articles describing how biomaterials can be used to either develop *in vitro* tissue models of gynecological tissue or designed as clinical interventions for gynecological diseases. Sutton and Shikanov provide an overview of how natural and synthetic biomaterials can support follicle development *in vitro* and how current 3D culture systems have improved our understanding of folliculogenesis. They then outline the current engineering challenges that must be overcome to develop an artificial ovary, which could restore fertility in survivors of pediatric cancers with premature ovarian insufficiency (POI). Next, a review by Gnecco details the biomaterials and *in vitro* culture systems that have been used to model the endometrium. They highlight the pros and cons of each approach and conclude by summarizing future limitations that must be addressed to continue to advance the field of reproductive health. The third article by Knight is a call to action for interdisciplinary research to address pelvic organ prolapse (POP), a condition currently affecting one in eight women. The previous synthetic meshes that were used had unacceptably high complication rates, leaving patients with POP with limited options. Thus, this article describes how biomaterial based clinical interventions could either promote tissue regeneration or provide mechanical support to pelvic organs.

### Reproductive organs—pregnancy

The third section of this Roadmap considers pregnancy and continues the theme of examining both *in vitro* research and possible future *in vivo* developments. Pregnancy is complicated by the co-existence of two (or more) genetically distinct individuals within the maternal reproductive tract. The maternal uterus interfaces directly with the fetal placenta and membranes; the fetus itself has no direct contact with the maternal tissues. There are ethical limits within the field of pregnancy research that have led to significant interest in the development of *in vitro* tools for studying the complexities of the maternal–fetal interface. In the first of two sections about placenta, Slaby and Weaver consider the need for biomimetic 3D materials systems that recapitulate placental architecture. They note that mechanistic studies of implantation and placental development would be enabled both for normal and pathological organ architectures. Continuing with the placenta, Hashemi considers placenta-on-a-chip microfluidic studies, which have permitted *in vitro* investigations of the placenta’s barrier function. These chip-based devices have provided opportunities to consider pharmacokinetics, an important consideration in pregnancy since drugs taken by the mother may be transmitted to the developing fetus. Returning the focus to maternal tissues, Zhang and House consider both *in vitro* and *in vivo* applications of novel silk-based biomaterials technologies to address the clinical problem of maternal cervical insufficiency in pregnancy. Current clinical practice involves insertion of a suture to keep the cervix closed until term birth; this practice has remained largely unchanged since the 1950s. The authors note that TE an *in vitro* cervix using a cell-seeded scaffold allows for study of the mechanisms of softening, while injectable hydrogel biomaterials present a novel and minimally invasive treatment paradigm for cervical insufficiency.

### Sex differences in other systems

The last section of this Roadmap details how biomaterials can be used to deepen our understanding of how biological sex differences affect non-gynecological tissues. Sex as a biological variable has been historically excluded from *in vitro* studies, animal studies, and clinical trials, resulting in a knowledge gap regarding how tissue properties change with regards to hormones and how specific treatments must be tailored to account for hormonal changes. This is still a relatively new field, as it was not until 2016 that the National Institute of Health began requiring sex as a biological variable to be included in study designs. Vogt and Aguado detail how including sex as a biological variable has brough to light sex dimorphisms in cardiovascular disease. They outline how biomaterials can play a unique role in characterizing sex-specific mechanisms, either by using 3D cell culture platforms to study mechanisms *in vitro* or designing biomaterial implants that take sex-specific tissue properties into account. Next, Bradford and Robinson provide a broad overview of how the field of TE has begun to account for sex as a variable and outline the current and future challenges. Specifically, they describe how biomaterial-based tissue engineered models of disease using sex-specific cells can fill the knowledge gap of how sex hormone signaling drives disease, tissue repair, and cell–material interactions. Third, Thomas and Lau review how hormone fluctuation during pregnancy and lactation directly bone mechanical properties. They point out existing gaps in knowledge and provide a framework for how understanding the effects of hormones on tissue material properties can be used to identify novel treatments for bone repair.

### Concluding remarks

Overall, these articles detail how interdisciplinary research combining basic science, engineering, biomaterials, and clinical research can have profound effects to improve the lives of patients that are currently drastically underserved. These article chapters represent a sample of a quickly evolving field and are not intended to provide an exhaustive review. However, they highlight different scientific and engineering approaches, different applications—both *in vitro* and *in vivo*—and illustrate the potential for further development of the research area. We hope that this Roadmap motivates researchers to engage with this important and understudied specialism as it emerges. Further, we hope this encourages funders and entrepreneurs to consider the possibilities in translating research into products designed to improve women’s lives worldwide.

## 2. Biomaterials based models of breast cancer

*Ning-Hsuan Tseng*^1^ *and Shelly R Peyton*^1,2^

^1^ Molecular and Cellular Biology Graduate Program, University of Massachusetts Amherst, United States of America

^2^ Department of Chemical Engineering, University of Massachusetts Amherst, United States of America

### Status

**Figure 1.**
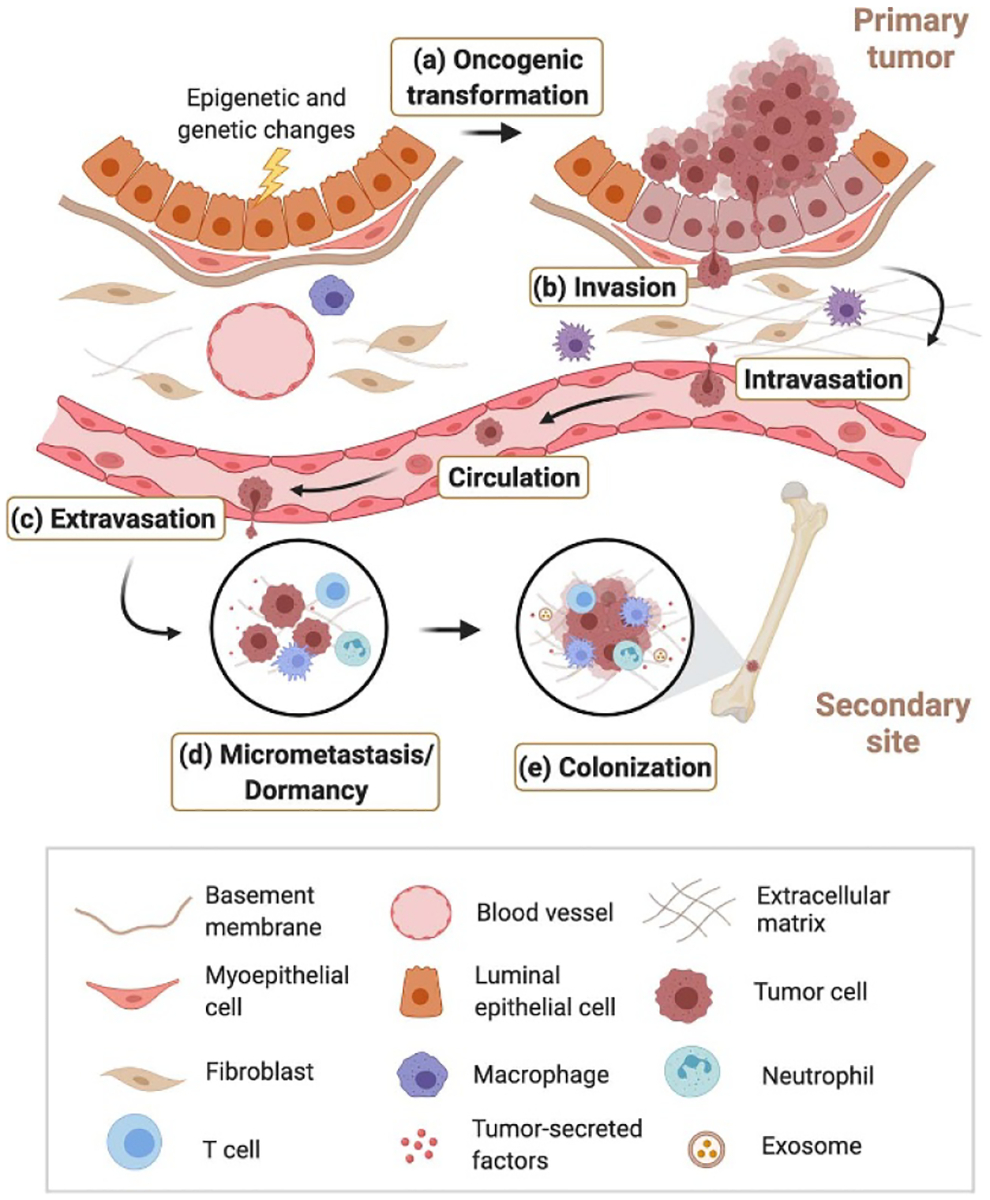
Breast cancer progression. Accumulation of epigenetic and genetic changes results in oncogenic transformation of epithelial cells (a). During progression, the ECM becomes stiffer, and cells invade through the BM (b), intravasate into the vasculature, survive in the circulation, extravasate (c), and establish a metastasis in a new microenvironment of a secondary site (d), (e). Created with BioRender.com.

Breast cancer progression is a multistep process, which often originates in the epithelium of the breast duct (figure 1). The breast microenvironment includes extracellular matrix (ECM) and multiple cell types (e.g. endothelial cells, fibroblasts (FBs), immune cells, and adipocytes). Normal mammary epithelial cell homeostasis is optimally supported by this environment. However, following oncogenic transformation, abnormal interactions within the microenvironment emerge (figure 1(a)). Breast tissue becomes progressively stiffer, and tumor cells become more contractile and respond to the changes in matrix compliance, leading to increased growth and invasion [12]. In the early stages of ductal carcinoma *in situ*, the basement membrane (BM) serves as a physical barrier, separating neoplastic cells from the surrounding stroma. As breast cancer progresses, an increase in the deposition of fibrotic ECM and dysregulation of tumor-stromal cell interactions leads to cancer cell invasion (figure 1(b)), intravasation into the vasculature, survival in the circulation, extravasation into a new tissue niche, such as bone, lung, liver, and brain (figure 1(c)). Some lesions expand rapidly, while others become arrested and remain dormant for several years until they outgrow and micrometastasize to form new tumors (figures 1(d) and (e)) [13]. The cellular, biochemical, and mechanical features of the TME have been incorporated into engineered biomaterials to study whether, and how, the TME prohibits, accelerates, or is a bystander of these steps of metastasis. In the early 1990s, a reconstituted BM derived from Engelbreth-Holm-Swarm mouse sarcoma, Matrigel, was the first such material system, used to demonstrate that proteins within, and the geometry of the ECM regulate the formation of 3D hollow-lumen acinar [14]. Since this initial model system, several more biomaterials have been developed as even more sophisticated models for breast cancer research, which we will discuss in this perspective.

### Current and future challenges

**Figure 2.**
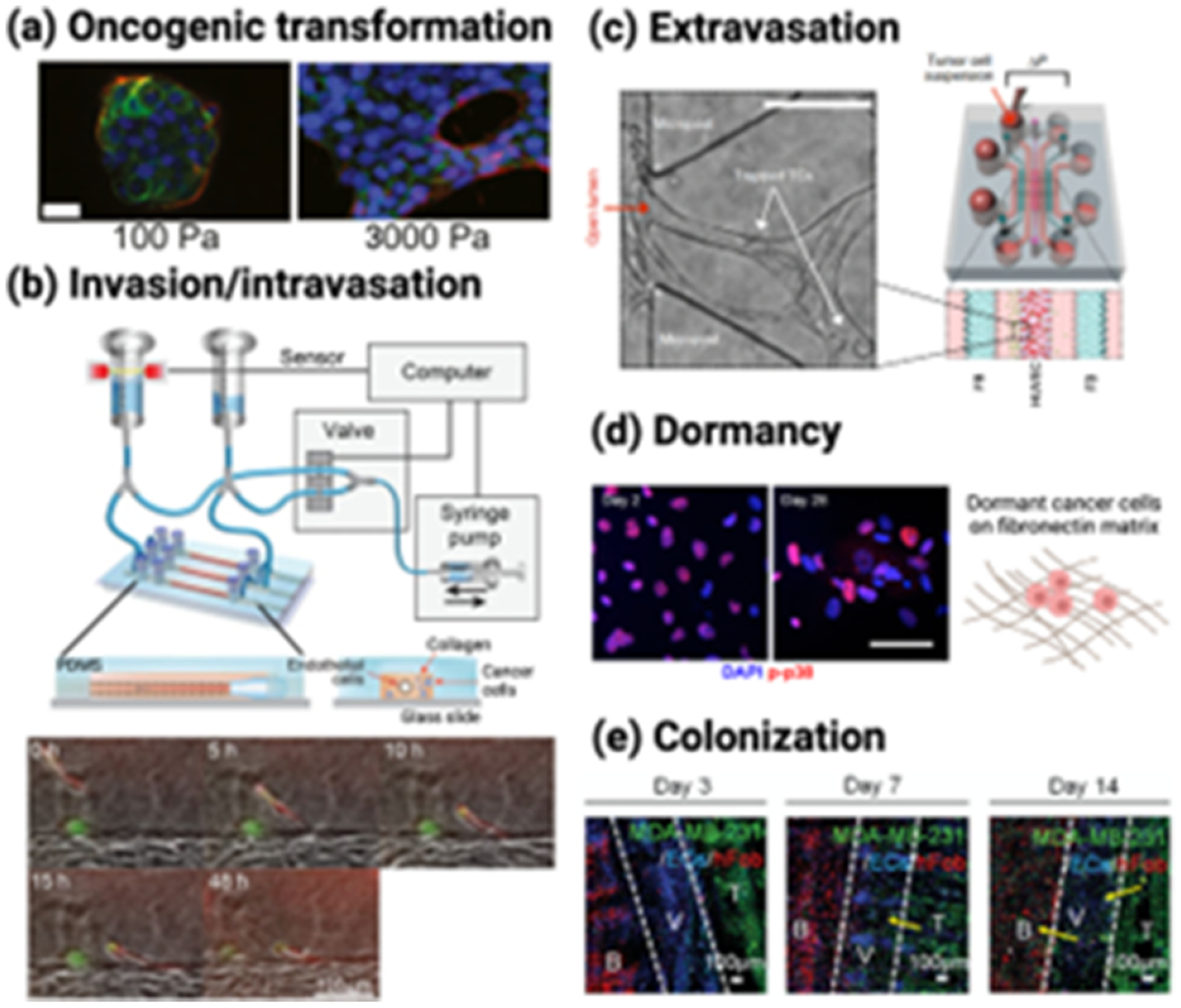
*In vitro* biomaterials-based breast cancer models to study metastatic progression. (a) MCF10A cells were grown on MeHA hydrogels that started compliant (100 Pa) and stiffened with UV light (3000 Pa) Fluorescent staining of Laminin (red), E-cadherin (green, epithelial-tomesenchymal transition marker), and nuclei (blue) shows that stiffening induced EMT. (b) Invasion/intravasation: cylindrical collagen channel lined with HMVECs within a PDMS housing perfused by a gravity flow system, with recycling using a solenoid valve and syringe pump. (c) Extravasation PDMS microposts spaced 100 *μ*m apart allowing HUVEC- and FB-laden hydrogels to form in the central (red) and side (green) regions, respectively Lumens form openings that connect the microvascular network to the media ports, allowing tumor cells (TCS) to be perfused via a hydrostatic pressure drop. (d) Dormancy: fluorescent staining for phospho-p38 (red) and DAPI (blue) in BCCson collagen after 2 and 28 d of serum starvation shows that BCCs are in a dormant-like state (e) Colonization: MDA-MB-231 cells metastasize toward bone over 14 d. The yellow arrows indicate the migration of invasive BOCs B: bone tissue, V: Vessel T: tumor tissue. Scale bars, 20 *μ*m (a): 100 *μ*m (b)–(e). [19] John Wiley & Sons. © 2021 Wiley-VCH GmbH.

*In vitro* biomaterial models have been developed to capture nearly every facet of the metastatic cascade (figure 2). Biomaterials from natural products such as collagen, alginate, and decellularized ECMs are ideal for including the biodiversity of the ECM, its fibrillarity, and protein–protein interactions. Materials made from synthetic polymers are a good alternative, offering more delicate control over microenvironment geometry, cell adhesion sites, stiffness, and porosity [15]. We outline current challenges here, that biomaterial models are beginning to tackle. Hypoxia: Tumor spheroids or organoids are increasingly popular 3D tumor models, which can recapitulate the cellular heterogeneity, cell–cell and cell–ECM interactions, and different cell layers within the TME. Due to their size, diffusion of nutrients and oxygen to the center of spheroids is limited, and they are excellent models for studying the effects of hypoxia on cancer progression. Hypoxia-inducible hydrogels have been synthesized from gelatin and ferulic acid, which crosslink by consuming O_2_ in a laccase-mediated reaction. These were used to model the effect of the O_2_ gradients on sarcoma cell invasion [16]. The next generation breast cancer models will include hypoxic organoids with these and other oxygen-sensitive materials. Organoids: Thus far, organoid models have been limited to Matrigel culture. An emerging and exciting area is combining more tunable biomaterials in which to grow these organoids, as well as use them to determine the minimalist set of ECM cues to support organoid growth. For example, Sachs *et al* developed primary and metastatic breast cancer organoids via BM suspension and found that most organoids were similar to the parental tumors with respect to their histopathology [17]. ECM heterogeneity: So far, most *in vitro* systems are specifically developed to give a spatially and temporally consistent, homogenous matrix. However, the ECM is spatially heterogeneous in all fibrotic tissues, including ECM surrounding and within breast tumors. To understand how heterogeneity affects cell phenotype in breast cancer, new models that can recapitulate the spatial and temporal changes of fibrosis are in development. We propose taking examples used in TE and applying them to studies of the TME. Spatially patterned stiffnesses of hyaluronic acid (HA) hydrogels showed that hepatic stellate cell activation could be predictably controlled [18]. Approaches such as this could be applied to determine how tumor progression is spatially regulated, as we elaborate on below.

### Advances in science and technology to meet challenges

Oncogenic transformation: Biomaterial models of breast cancer have been created to capture ECM changes during nearly every step of the metastatic cascade (figure 2). To understand how this matrix stiffening around a primary tumor can impact oncogenic transformation (namely, the epithelial to mesenchymal transition, EMT), methacrylated glycosaminoglycan HA (MeHA) hydrogels were created that could stiffen on demand in response to UV light (figure 2(a)). Upon matrix stiffening, mammary epithelial cells (MCF10A) lost their epithelial characteristics and gained a mesenchymal morphology, mimicking the correlation between ECM stiffness and EMT observed *in vivo* [20]. Invasion/intravasation: Microfluidic devices include a vasculature into biomaterials via channels and continuous cell or medium perfusion. Wang *et al* created a type I collagen hydrogel scaffold with a cylindrical channel lined with a monolayer of human dermal microvascular endothelial cells (HMVECs). Dual-labeled breast cancer cells (BCCs) MDA-MB-231 (GFP: nuclei, RFP: cytoplasm) were embedded within the collagen matrix. Then, BCCs could be observed as they invaded through the collagen ECM (figure 2(b)) and intravasate into the artificial vessel [21]. Extravasation: Another PDMS microfluidic device with perfusable microvascular networks for real-time quantification of BCC extravasation was built by separately injecting human umbilical vein endothelial cells (HUVECs) and human lung FBs suspended in fibrin gels into the central and side gel regions (figure 2(c)) [22]. Dormancy: Following extravasation, some tumor cells remain dormant for years or decades, and this has been challenging to model *in vitro*. Barney *et al* established a cell culture system with controlled ECM proteins and serum deprivation to examine BCCs entering and exiting dormancy. A subset of BCCs expressed active p38 after 28 d of serum deprivation which aligned with dormancy characteristics described *in vivo* (figure 2(d)) [23]. Colonization: For studying metastatic growth in the bone cavity, Cui *et al* developed an *in vitro* vascularized tissue model using stereolithography 3D printing with photocurable inks prepared by mixing gelatin-based ink gelatin methacrylate (GelMA), polyethylene glycol (PEG) diacrylate ink, and nanohydroxyapatites at various ratios to obtain bone, tumor, and vessel channel chambers with different biomechanical properties. The human fetal osteoblasts and MDA-MB-231 cells were seeded onto the bone and the tumor matrix, respectively. HUVECs were injected into the vessel where BCCs metastasized toward bone over 14 d (figure 2(e)) [24]. Although models have been made to study these individual steps of cancer metastasis, we still cannot recapitulate the entire cascade in a single *in vitro* system, nor the dynamic transitions between steps. We suggest this as a grand challenge for the materials community.

### Concluding remarks

With advances in designs and technologies, engineered *in vitro* breast cancer models have become more complex thus can better mimic *in vivo*-like TME. Applying 3D bioprinting and microfluidic devices allows spatial controls on the biochemical compositions, cells, and vasculature structures. These models are used to study cell–cell and cell–ECM interactions, signaling pathways related to tumor malignancy, drug screening, etc. Based on the specific aspect of the metastatic cascade they are studying, researchers can choose from various breast cancer models of different complexity and functionality. One remaining challenge is our inability to compare results across models. In addition, tumor heterogeneity both within and between patients makes patient-specific drug screening and predicting outcomes difficult, even with the simplest model systems. Future work must combine patient-derived organoids with innovative biomaterials-based models, likely with the ability to incorporate immune cells. Regardless, collaborations between cancer biologists and bioengineers is enabling rapid increases in technology development.

### Acknowledgments

This work was supported by an NSF CAREER (DMR1454806), an NIH Grant R21CA223783, and a grant from the Jane Koskinas Ted Giovanis Foundation for Health and Policy awarded to SRP.

## 3. Biomaterials in nipple areolar reconstruction

Pieper Holeman, Shannon Mc Loughlin and John P Fisher

Fischell Department of Bioengineering, University of Maryland, College Park, MD, United States of America

### Status

Approximately one in eight women will be diagnosed with breast cancer [25], making it an extremely prevalent disease. The current standard treatment for early (stages I and II) and mid-stage (stage III) patients is surgical intervention paired with radiation or chemotherapy treatment [25], with the most common surgical techniques being breast-conserving surgery (BCS), mastectomy, and nipple-sparing mastectomy (NSM). Early-stage breast cancer diagnoses are treated using BCS and NSM techniques, which remove cancerous tissue and the surrounding area while preserving some breast tissue and NAC. As the cancer progresses to mid-stage, mastectomies become the most common procedure, occurring in 68% of diagnoses [25]. Mastectomies completely remove the breast tissue and the NAC, with reconstructive surgery following shortly after. Although other procedures result in better cosmetic outcomes and reduced psychological burden, patients are increasingly electing for a total mastectomy due to a lower chance of cancer recurrence, potential avoidance of adjuvant therapy, and desire for symmetry [25–27].

Although mastectomies are life-saving procedures, there are significant adverse sequelae that follow the procedure. Losing one or both breasts can have negative psychological and social impacts on breast cancer patients. Many patients experience significant pain, decreased strength, fatigue, sleep loss, and depression post-treatment [26]. BRS can help restore the breast after a mastectomy and has been found to mitigate post-treatment trauma and improve body image, self-confidence, and psychosocial well-being [28]. However, it has been reported that breast satisfaction decreases over time in reconstruction patients [29]. Patients that elect for NAC reconstruction surgery, in addition to BRS, reported higher levels of emotional satisfaction than patients without reconstructive surgery, due to the restoration of the critical visual structure. However, one study found that common areas of dissatisfaction with NAC reconstruction are lack of projection, pigmentation, and natural-looking appearance [28]. Therefore, there is a significant need to develop NAC reconstruction techniques to provide a long-term positive outcome.

### Current and future challenges

Current NAC reconstruction techniques report high rates of initial patient satisfaction; however, long-term satisfaction rates are relatively low. This decrease in satisfaction is due to flattening of the nipple projection and recurring surgeries needed to maintain the reconstruction [28–30]. NAC reconstruction techniques include tattooing, alloplastic, and autologous reconstruction [29–31]. Tattooing is considered the most visually accurate form of reconstruction; however, it is simply cosmetic and does not produce an anatomical projection. Various alloplastic materials have been used in NAC reconstruction, including calcium hydroxyapatite, HA, silicone gel, and porcine small intestinal submucosa. Autologous reconstruction techniques use vascularized skin muscle, skin flap suturing, cartilage, or fat tissue to build a projected skin tab [30, 31]. However, it is reported that 1–2 years after surgery, NAC projection loss ranges between 43% and 59% and requires additional procedures to maintain the cosmetic appearance [31]. NAC reconstruction techniques continue to be a clinical challenge and can benefit from a regenerative tissue approach.

Existing NAC reconstruction techniques often cannot accurately capture the NAC’s appropriate size, shape, and tactile properties. Current TE approaches to create lasting NAC projection utilize decellularized matrices, dermal grafts, and bioprinted scaffolds [32, 19]. Decellularized NAC scaffolds implanted in non-human primates have shown similar epithelial coverage and vasculature to native tissue after 6 weeks post-engraftment. The projection, shape, color, and sensitivity characteristics of decellularized NAC scaffolds have not yet been reported [32]. Bioprinting allows for the generation of personalized implants fabricated from biocompatible materials and living cells, using an additive manufacturing approach. Recently, bioprinting has been utilized to generate scaffolds in the complex anatomy of the NAC. One approach integrated methylcellulose (MC) and PEG networks with a GelMA bioink. The MC-PEG architecture provided the structural support necessary to resist projection flattening and mirrored the mechanical properties of natural NAC tissue. The biodegradable GelMA allowed for cellular infiltration and dermal regrowth. A 4 week *in vivo* study demonstrated promising results for biocompatibility and shape retention, as there was a mild local foreign body response, minimal fibrotic encapsulation, evidence of neovascularization, and NAC structural maintenance in explanted constructs [19]. Both bioengineered approaches have shown encouraging preliminary data; however, long-term studies are needed to truly measure the potential of these approaches for NAC reconstruction.

### Advances in science and technology to meet challenges

Current bioengineered implants for NAC reconstruction still have challenges that need to be addressed. After NAC reconstruction, the major reasons for dissatisfaction were attributed to the lack of projection, color, and natural appearance [28]. Sustaining nipple projection is incredibly challenging due to the contraction forces of healing skin post-surgery. The MC-PEG architecture of the bioprinted scaffold resists these forces and may result in a lasting projection. Future work needs to examine the incorporation of biocompatible materials to enhance NAC scaffold biological properties, without compromising mechanical strength.

Pigmentation is an incredibly significant aspect of NAC reconstruction and, like projection, is challenging to sustain. The only permanent method of creating pigmentation is tattooing; however, patients must wait several months for the skin to heal post-mastectomy and it is preferred that patients do not undergo further reconstruction surgeries after tattooing. While there are a variety of commercial skin grafts that can produce pigment in skin reconstructions through manipulation of melanocyte culture, these strategies have yet to be applied to NAC reconstruction. Future designs of bioengineered NAC grafts will likely take inspiration from current artificial skin grafts to produce lasting NAC pigmentation.

When considering the translational capacity of NAC implants to patient bedsides, ease of manufacturing must be considered. While decellularized tissues offer significant biological advantages for regenerative approaches, the processes of production require complex protocols that often generate inconsistent results, regarding cellular content removal. Additionally, the donor tissue limits the size and shape of decellularized scaffolds, resulting in cosmetic inconsistencies for NAC reconstruction. Additive manufacturing techniques combat this issue through high-throughput design and fabrication of more patient-specialized constructs. Additionally, the scaffolds are designed computationally, and therefore many iterations can be manufactured to create more cosmetically similar scaffolds to fit a range of patients.

### Concluding remarks

While mastectomies provide breast cancer patients with a sense of security regarding the chances of cancer recurrence, these procedures can often cause significant emotional and psychological damage to patients. Many women experience serious emotional trauma when observing their mastectomy scars. Breast reconstructive surgeries can mitigate some of these issues, but many women still face negative psychological outcomes due to the lack of the critical nipple anatomy. NAC reconstruction techniques have been shown to mitigate post-treatment traumas and improve body image, self-confidence, and psychological well-being. However, current clinically available reconstruction methods lack the ability to sustain the nipple projection and pigmentation. In order to achieve both the visual and tactile properties of native nipple tissue, regenerative TE approaches must be considered. While current TE strategies have generated promising preliminary results, future solutions must focus on maintaining nipple projection, mimic natural pigmentation, and be easily fabricated.

### Acknowledgment

We would like to acknowledge support from the NIH/NIBIB Center for Engineering Complex Tissues under Grant No. P41EB023833.

## 4. Biomimetic materials for ovarian research

*Allison Sutton*^1^ *and Ariella Shikanov*^1,2^

^1^ Department of Biomedical Engineering, University of Michigan, Ann Arbor, MI 48109, United States of America

^2^ Program in Cellular and Molecular Biology, University of Michigan, Ann Arbor, MI 48109, United States of America

### Background

Pediatric cancer survival rates have increased significantly over the past few decades, reaching an estimated 84% in the United States [33]. However, anticancer treatments such as chemotherapy and radiation are known to have profound gonadotoxic effects, leaving female patients at risk of POI, which can cause long-term loss of fertility, disruption of the endocrine signaling vital for normal growth and development, and early menopause. Options to preserve fertility and maintain normal endocrine function in pediatric patients are limited; prepubertal girls are not candidates for fertility preservation via ovarian stimulation followed by embryo or oocyte cryopreservation, which is commonly employed for adult patients, and hormone replacement therapy (HRT) to initiate puberty and maintain long-term endocrine function is limited in its ability to fully replicate the complex and varying hormone levels of natural physiology. The American Society for Reproductive Medicine practice committee recently removed the experimental label from ovarian tissue cryopreservation and autotransplantation [34], which offers a new and promising option to restore both fertility and endocrine function, but for patients with blood borne or metastatic cancers, autotransplantation presents a risk for reintroducing malignant cells and is thus not appropriate. Novel approaches in biomaterials’ design aim to address these gaps in treatment availability and restore fertility and endocrine function to survivors of pediatric cancers with POI. Specifically, this section will provide an overview of novel biomimetic systems for the culture of ovarian follicles *in vitro*, the development of an artificial ovary, and immunoisolating systems for restoration of endocrine function.

### *In vitro* culture systems to promote folliculogenesis

The follicle is the structural and functional unit of the ovary, composed of an oocyte surrounded by layers of supportive somatic cells. The native ovary contains a finite reserve of inactive primordial follicles, which sequentially activate, grow and mature in a process known as folliculogenesis. Granulosa cells in the activated follicles proliferate to form multiple layers surrounded by a BM and theca cells. The follicle continues maturation through the antral stage and oocytes resume meiosis prior to ovulation, pausing in the second meiotic metaphase (MII). Depending on the species, either a single or multiple follicles will complete ovulation, leaving behind the remaining granulosa and theca cells to form the corpus luteum structure. Successfully replicating this process *in vitro* could enable the production of mature oocytes for fertilization from fresh or cryopreserved immature ovarian tissue samples, providing pediatric cancer patients with a pathway for fertility preservation.

*In vitro* folliculogenesis has held the interest of researchers for decades, beginning with whole tissue culture of mouse and rat ovaries on watch glass by Martinovich [35]. Two-dimensional (2D) cultures of isolated follicles were first pioneered by Eppig [36], and these systems have seen limited success with secondary follicles from murine species. However, in cultures of early-stage follicles or those from larger animal species, the granulosa cells quickly migrate onto the surface away from the oocyte, arresting its growth [37]; later work by Eppig in culturing primordial murine follicles as a whole organ culture followed by the isolation and culture of oocyte-granulosa cell complexes resulted in live-birth but was limited by a low yield [38]. It is now well-understood that paracrine and autocrine signaling between the oocyte and its surrounding cells is vital to folliculogenesis, especially in the early stages of growth. This complex regulatory process necessitates the use of culture systems capable of maintaining the 3D structure and signaling interactions within the follicle.

Alginate was the first biomaterial utilized for hydrogel-based 3D culture of ovarian follicles. It is a natural polysaccharide derived from algae and is a popular material for a wide variety of biomedical applications. Its mild gelation process and pore size permissive for diffusion of oxygen and nutrients is well-suited for encapsulation of follicles for culture. Individually encapsulated secondary murine follicles survive and develop readily in alginate, and oocytes can be retrieved and fertilized to produce viable embryos and fertile offspring [37]. Yet, translation of alginate-based culture systems to follicles from humans or large animals has been limited due to follicles’ inability to degrade the alginate matrix and expand; due to lack of degradation, alginate exerts increasing compressive force on the follicle as it grows. Murine follicles that only grow to 350 *μ*m in diameter were not affected, but human follicles and those from other large species expand significantly more, reaching a few millimeters in diameter at antral stages, which calls for a degradable material or culture methods that do not exert a compressive force.

So far, mature MII oocytes have only been obtained from human tissue using multi-step methods, in which follicles are cultured in different environments across the stages of folliculogenesis. In a 2015 study, researchers encapsulated secondary human follicles in alginate and cultured them for 10–15 d until they reached the antral stage, then released the follicles from the hydrogels and cultured them in low-attachment plates for up to 40 d before performing *in vitro* maturation to obtain MII oocytes [39]. Other groups have obtained MII oocytes by first utilizing tissue culture of ovarian cortex samples, in which early-stage human follicles can grow within their native environment, before dissecting out larger follicles for individual culture in V-bottom culture plates [40] or ultra-low attachment microplates [41]. While promising, these multi-step methods are labor-intensive, requiring significant handling of the delicate follicle across steps. As a result, the most pressing goal is to design a single-step culture system based on degradable biomimetic materials that support follicle growth through the complete process of folliculogenesis, particularly for the culture of human follicles.

**Figure 5.**
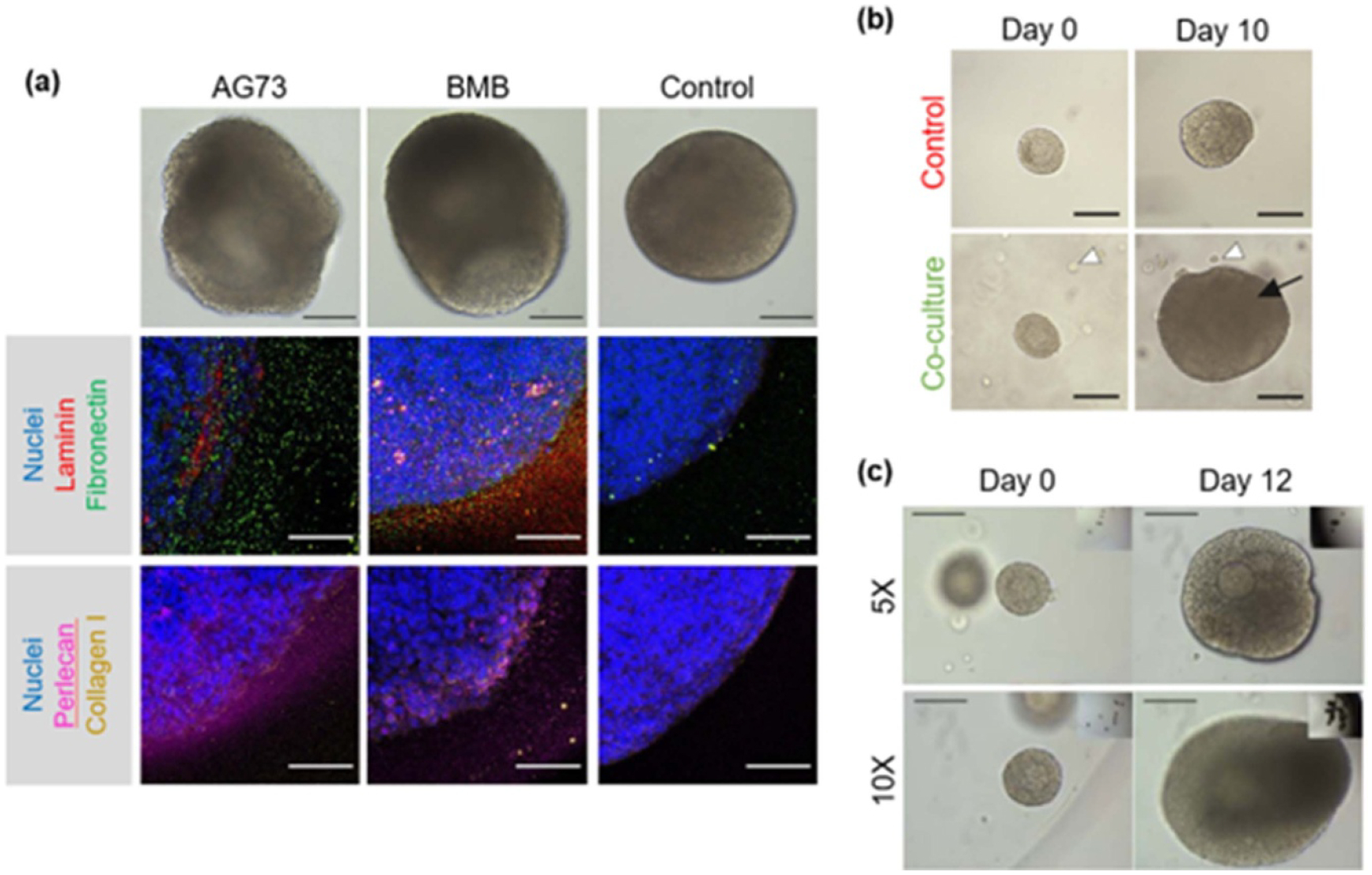
Systems for *in vitro* folliculogenesis. (a) PEG gels modified with ECM-se questering peptides AG73 and BMB support follicle maturation and successfully retain cell-secreted ECM after 10 d in culture. (Top) Representative brightfield images of follicles. (Middle) Immunostaining of follicles for fibronectin (green) and laminin (red) with nuclear counterstain (blue). (Bottom) Immunostaining of follicles for perlecan (magenta) and collagen 1 (yellow) with nuclear counterstain (blue). Scale bar = 100 *μ*m (top), 50 *μ*m (middle, bottom). Reprinted from [43], Copyright (2021), with permission from Elsevier. (b) ADSC Co-culture improves follicle growth over 10 d of culture. (ADSCs (white arrowheads), darkening of granulosa cells before antrum formation (black arrow)), Scale bar = 100 *μ*m. Reproduced from [47] with permission from the Royal Society of Chemistry. (c) Multiple follicle culture in groups of 10X supports improved growth of primary follicles compared to groups of 5X over 12 d of culture. Scale bar = 100 *μ*m. Reproduced from [49]. CC BY 4.0.

One example of such biomimetic degradable material is PEG, a highly tunable polymer that provides a myriad of opportunities for engineering the follicle environment with a high level of control. PEG gels modified with proteolytically degradable crosslinkers have been established to support the survival and growth of secondary murine follicles, and initial studies showed that the material accommodated the volumetric expansion of growing follicles through degradation of the surrounding gel [42]. More recently, this system has been further customized to incorporate ECM-sequestering peptides in the hopes of capturing ECM molecules and growth factors naturally secreted by follicles to restore some of the native regulatory functions of the follicle microenvironment (figure 5(a)). The hydrogels successfully retained cell-secreted ECM, supported increased growth of early secondary follicles, and exhibited increased concentrations of folliculogenesis-regulating cytokines in the culture media [43]. In the future, PEG-based culture systems may be modified with additional bioactive components to further support the full process of folliculogenesis *in vitro*.

The physical and biomechanical properties of the *in vitro* culture system constitute only one aspect of the bioengineering design. Another critical component of the *in vitro* culture is the reciprocal crosstalk between the follicles and the extrafollicular cells in the ovary. The inability to recreate the complex paracrine signaling that occurs in the early stages of folliculogenesis leads to prohibitively low yields of mature fertilizable eggs from early-stage follicles cultured *in vitro*. Ongoing work is investigating culture methods that can support early-stage follicles, which make up the majority of the follicle reserve. Several cell types have been investigated for use as ‘feeder cells’ that could provide necessary secreted factors, including murine embryonic FBs [44], ovarian stromal cells [45], and adipose-derived stem cells (ADSCs) [46, 47] (figure 5(b)). While these systems have found some success, researchers often face challenges in designing culture conditions to adequately support multiple cell types, and feeder cells do not fully recreate the signaling environment of the native follicle. Alternatively, multiple follicle culture has shown promise; alginate hydrogels containing primary murine follicles cultured in groups of ten support significantly improved growth and survival [48] (figure 5(c)). These culture systems also enable further interrogation of the signaling processes governing early-stage folliculogenesis, revealing synergistic cross-talk between follicles and unique transcriptional signatures [49, 50]. Future work in this area will aim to elucidate the unique needs of primordial and primary follicles in culture and better support their growth through the addition of exogenous factors.

Recently, researchers in the area of organ-on-a-chip technologies have utilized microfluidics to explore the impact of dynamic culture conditions on the ovary. One of the applications showing the most promise is in oocyte maturation and fertilization. In one study, murine cumulus-oocyte complexes, matured *in vitro* under flow conditions reached the MII phase in significantly higher proportions than those in static conditions [51]. Oocytes from both groups were then fertilized, and those that had been matured under dynamic conditions had higher rates of fertilization and blastocyst formation. The researchers concluded that the dynamic culture conditions may benefit the maturing oocytes by disrupting oxygen gradients at the cell surface, more closely mimicking the low-oxygen native environment and protecting against damage from reactive oxygen species.

Other microfluidic systems are being developed to support folliculogenesis more broadly. In a 2017 study, researchers sought to model the 28 d human menstrual cycle via a microfluidic platform containing murine ovarian tissue and downstream human fallopian tube, endometrium, ectocervix, and liver tissue [52]. In response to exogenous follicle-stimulating hormone and luteinizing hormone (LH), controlled via pneumatic actuation, the ovarian tissue underwent follicle growth, maturation, ovulation, and granulosa cell luteinization. The tissue also produced steroid hormones in human-like patterns and at higher levels than seen in static culture, illustrating the potential benefits of microfluidic technology in modeling human reproductive tissue. However, these systems face the same challenges as other *in vitro* platforms in supporting ovarian tissue from larger species. In another study, individual follicles and ovarian cortex tissues from domestic cats and dogs were cultured in alginate within a microfluidic channel [53]. Follicles from both species were supported in short-term culture under flow conditions in the device, but with no improvement over conventional static culture. The system also illustrated species-specific differences, as cortical tissues from domestic dogs generally responded more poorly to culture conditions compared to cat tissues, highlighting the challenges remaining in adapting *in vitro* culture systems for large mammalian species.

As more is discovered about the complex needs of ovarian follicles in each stage of growth, precise engineering of the follicle microenvironment, including both a supportive matrix with the capability to undergo cellular remodeling and carefully formulated culture conditions, will enable the movement towards successful culture of immature human follicles for fertility preservation.

### Engineering approaches for *in vivo* ovarian function

**Figure 6.**
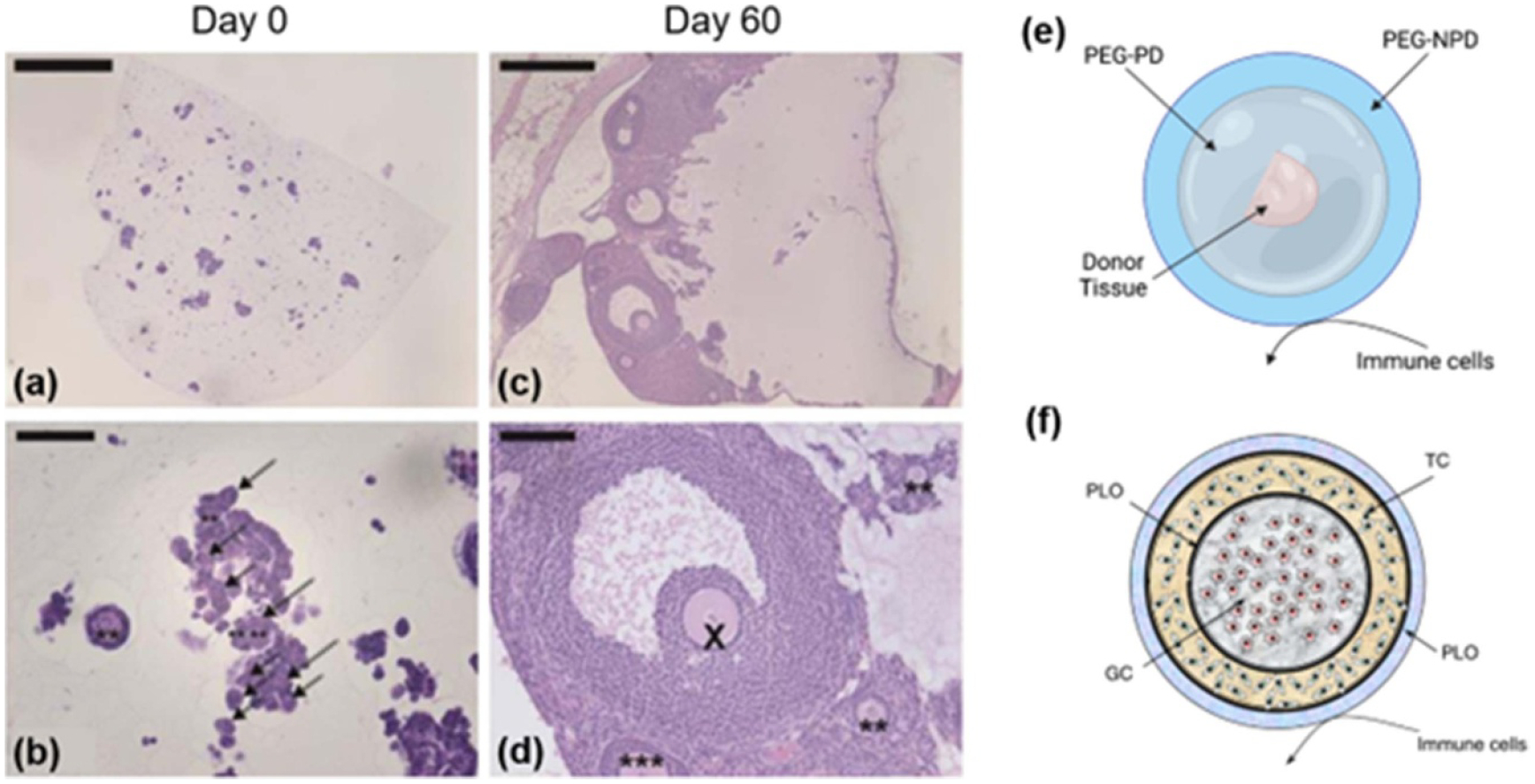
*In vivo* systems for the restoration of ovarian function. (a)–(d) Folliculogenesis in a PEG-based artificial ovary. Over 60 d, follicles developed from (a), (b) a population of primordial and primary follicles to (c), (d) a population containing multiple antral follicles. (Primordial (dotted arrow), primary follicle (**), secondary follicle (***), antral follicle (X)), scale bar = 500 *μ*m (a), (c), 50 *μ*m (b), 100 *μ*m (d). Reproduced from [56]. CC BY 4.0. (e), (f) Schematics of immunoisolating capsule systems for the restoration of endocrine activity. (e) Donor tissue encapsulated in a proteolytically degradable (PEG-PD) core surrounded by a non-proteolytically degradable (PEG-NPD) shell. Created with BioRender.com. (f) Granulosa cells (GCs) and theca cells (TCs) encapsulated in a multilayer alginate capsule separated by poly-L-ornithine (PLO) for cell hormone therapy. Reproduced from [59]. CC BY 4.0.

In the future, TE approaches may enable the creation of an artificial ovary, which would provide both fertility and endocrine function to patients with POI. A number of materials, including alginate and other natural materials such as fibrin [54], have been explored for this purpose and have been reviewed elsewhere [55]; however, many of the same design requirements for *in vitro* culture systems apply, making tunable and degradable artificial materials such as PEG an attractive choice. In a 2016 study, a PEG-based artificial ovary containing mainly early-stage murine follicles was implanted in ovariectomized mice for up to 60 d (figures 6(a)–(d)). Estrous cycles resumed and follicle stimulating hormone (FSH) levels were reduced to pre-ovariectomy levels by day 49, and the hydrogel supported the survival and development of the follicles, with increasing proportions of secondary and antral follicles observed across timepoints. By 60 d post-implantation, primordial and primary follicles made up 60% of the follicle pool, representing some maintenance of the immature follicle reserve present in the native ovary [56]. This and other studies represent promising systems for the development of the artificial ovary. However, fully engineering the ovary will require significant developments in the understanding of this highly complex organ.

In addition to the challenges in recreating the full folliculogenesis process faced *in vitro*, an artificial ovary would require the long-term maintenance of function. Much is still unknown about the mechanisms governing follicle activation, a vital consideration for the design of implants that must support long-term maintenance of the follicle reserve. Additionally, translation for human use would require investigation of the ability of tissue-engineered systems to recreate the dominant follicle selection that occurs in the late stages of human folliculogenesis, as well as the complex and changing endocrine role of the ovary across the human reproductive lifespan. A bioengineered artificial ovary would represent the full combination of the field’s knowledge of activation and folliculogenesis, ovulatory function, and the role of the ovary in the human endocrine system, and much remains to be learned before its translational potential can be realized.

Until it is possible to fully engineer the ovary and restore fertility and ovarian endocrine function, an *in vivo* solution for endocrine function could be combined with *in vitro* fertility preservation methods to fully address patient needs. Given the limitations of HRT, the ability to restore natural endocrine activity would represent a major improvement in clinical options for patients facing diminished ovarian function. One possibility is the use of donor ovarian tissue; however, current organ or tissue transplantation requires the use of immunosuppressive therapies to prevent rejection, limiting the clinical adoption of ovarian tissue transplantation. Our group has pioneered the design of an immunoisolating capsule for the transplantation of ovarian allografts (figure 6(e)). The capsule utilizes a dual PEG system and is comprised of a proteolytically degradable core surrounded by a nondegradable shell. In a 2019 study, dual capsules implanted in ovariectomized immune competent mice supported the survival of allograft tissue, enabled the resumption of estrous cycles and endocrine function, and protected the implant from immune system activity [57]. Ongoing work aims to adapt this system for human applications by addressing the metabolic needs of larger human tissue samples while maintaining the immunoisolating properties of the capsule. Alternatively, isolated rat granulosa and theca cells have been encapsulated in layers of alginate, separated and surrounded by poly-L-ornithine, to create a cell hormone therapy system [58] (figure 6(f)). These microcapsules have been shown to survive and secrete hormones for up to 90 d in an ovariectomized rat model [59]; recently, the estrogen production of the system was further improved with the incorporation of bone marrow-derived stem cells [60]. These capsule systems represent highly translational possibilities for the restoration of endocrine activity in patients facing POI.

### Concluding remarks

The activation, development, and function of the ovarian follicle is an immensely complex and highly regulated process about which much remains to be understood. As the knowledge of the field has advanced, biomaterials have come to represent a key part of the engineering solutions being investigated for patients facing diminished ovarian function following cancer treatment. Natural and synthetic hydrogels provide supportive culture environments that can enable the development of follicles *in vitro* and improve the field’s understanding of folliculogenesis. Future progress using degradable materials such as PEG may enable the culture of human follicles *in vitro* through the full process of folliculogenesis, representing a major advancement in assistive reproductive technology and providing a previously inaccessible option to certain cancer patients desiring fertility preservation. As more is discovered about the requirements and roles of the human ovary, biomaterials may enable the engineering of an artificial ovary to support both the fertility and endocrine needs of patients with POI. Until then, *in vivo* systems such as the immunoisolating capsules discussed here could restore the endocrine function that cannot be fully recreated by HRT and, in combination with *in vitro* strategies for fertility preservation, address the clinical gaps currently faced by survivors of pediatric cancer.

### Acknowledgment

National Institutes of Health Grants R01HD104173, R01HD09823, R01 HD099402.

## 5. Biomaterials for endometrial research

Juan S Gnecco

Department of Biomedical Engineering, Tufts University, Medford, MA, United States of America

### Status

The endometrium is the mucosal lining of the uterus and the tissue responsible for the establishment and maintenance of pregnancy. It is a complex and dynamic tissue that undergoes morphological and biochemical changes in response to sex hormones resulting in cyclical events of growth, differentiation, and shedding [61]. However, our understanding of the cellular, molecular, and biophysical mechanisms that mediate both endometrial reproductive function and dysfunction remains elusive. This is in part due to the lack of physiologically relevant model systems that dissect endometrial physiology and recapitulate the human condition. Implementing TE principles to the field of gynecology has the potential to transform women’s reproductive health by providing innovative tools that mechanistically dissect endometrial biology and the pathophysiology of endometriotic diseases. This short review discusses the recent advances in biomaterials and engineered *in vitro* model systems as emerging platforms that enable the dissection of fundamental spatial and temporal processes in human endometrium with the goal of identifying novel therapeutic targets for reproductive disorders.

### Current and future challenges

**Figure 7.**
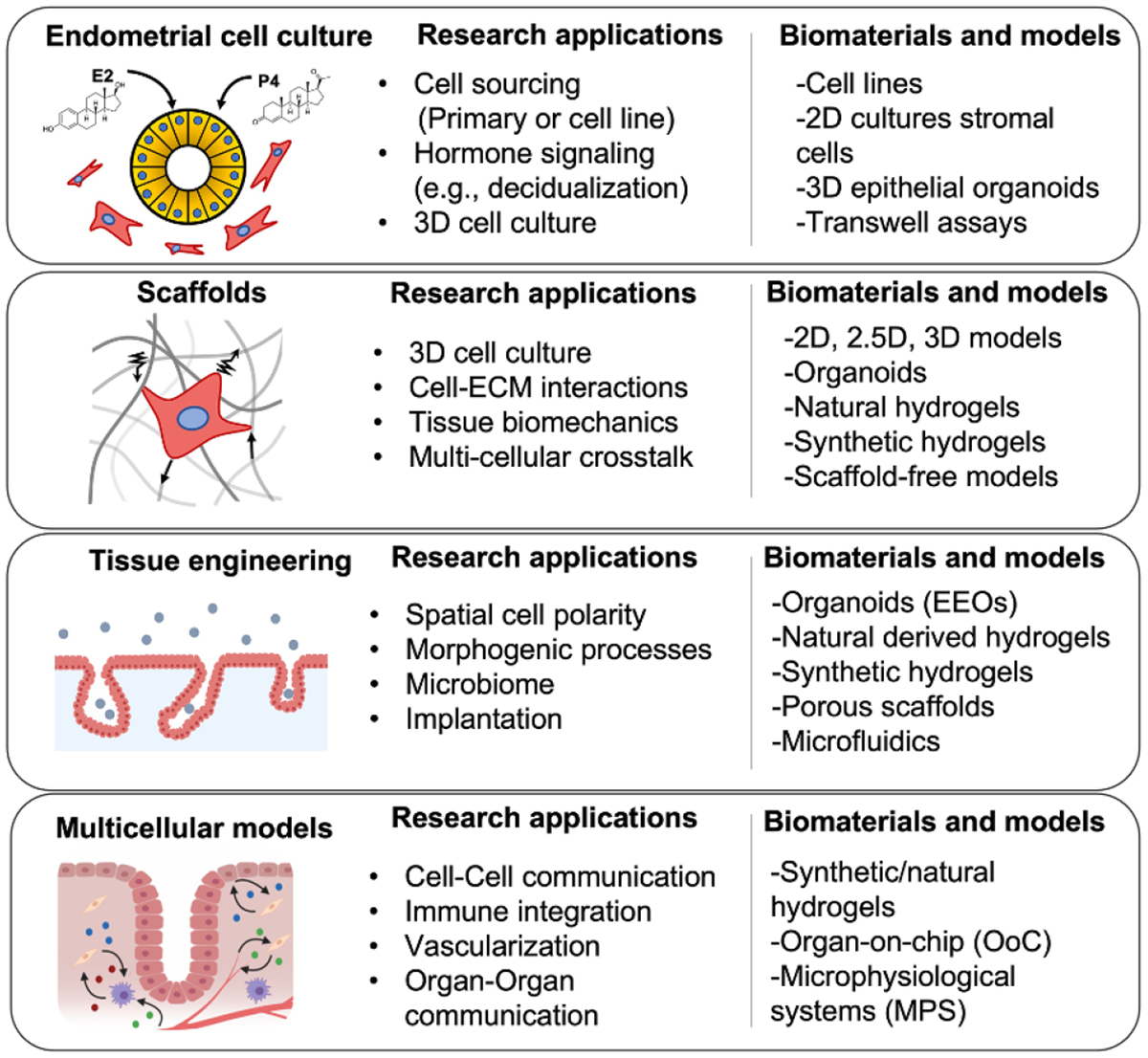
Strategies for modeling the human endometrial reproductive function *in vitro*. Workflow for cell culture techniques, scaffolds, TE, and multicellular model, their relevant biological applications, and examples of the types of models used to address those needs.

**Figure 8.**
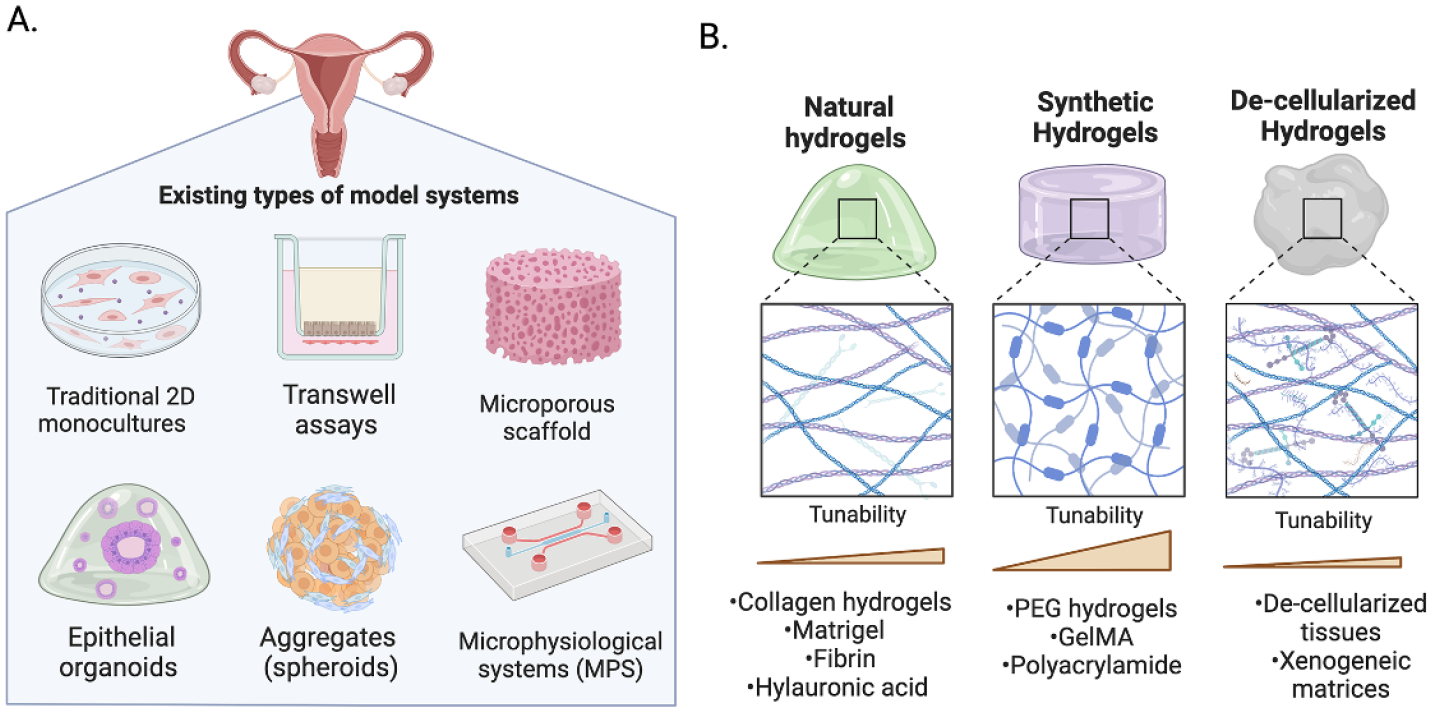
Existing and emerging *in vitro* tools for modeling the endometrium. (A) Schematic of the existing types of models available to model the endometrium *in vitro*. (B) Overview of existing hydrogel biomaterials for EEO cultures [62, 66, 74]. Created with BioRender.com.

Historically, the gynecological field has always been challenged by the ability to reproducibly culture, maintain, and expand primary cells derived from human donors, especially for the endometrial epithelia. This has severely limited the exploration of epithelial cell biology and made the field reliant on cell lines. Moreover, the 2D cell models using traditional tissue culture plastic (polystyrene) that are widely used for *in vitro* studies often lack physiologic components of the native tissue microenvironment including its structural, cellular, and biophysical properties. The recent development of 3D endometrial epithelial organoids (EEOs) are a particularly tractable tool to investigate, propagate and expand patient derived epithelial populations and represent a revolutionary step in overcoming these challenges [62, 63]. These 3D cell culture systems are poised to advance the field; however, several technical challenges remain to fully take advantage of these technologies. Specifically, the reliance on sarcoma-derived hydrogels like Matrigel as the gold-standard biomaterial to generate organoids limits its reproducibility and translational potential. Matrigel’s ill-defined heterogenous composition, variable xenogeneic contamination and limited tunability make it challenging to model other cellular and biophysical components of the endometrial microenvironment. Hormone response in the endometrium, and subsequent function, is mediated by numerous signaling pathways including cell–cell communication, cell–ECM interactions, and biomechanical cues; therefore, technologies that incorporate these additional cell populations and enable the interrogation of these dynamic and physiological processes in the human endometrium are desperately needed (figure 7). Advances in biomaterials, cell culture methodologies and platforms offer an exciting opportunity to address these needs and dissect the fundamental biological processes that regulate reproductive health. Several existing and emerging approaches include, but are not limited to, synthetic or natural hydrogels embedded multi-cellular cultures [64–66], scaffold-free aggregates (spheroids) [67, 68], porous scaffolds [69], organ-on-chip (OoC) technologies [70, 71] and multi-organ microphysiological systems (MPS) [72, 52] (figure 8(A)). Herein, hydrogel biomaterials are discussed in detail as a key enabling technology to establish organoid and multi-cellular *in vitro* model systems to study the endometrial microenvironment.

### Advances in science and technology to meet challenges

Addressing the challenges mentioned above requires implementation of engineering principles to deconvolute the complexity of the endometrial microenvironment. Current approaches to generate 3D models of the endometrium utilize both natural-derived and synthetic polymers to make hydrogels [64, 65] and porous scaffolds [69]. These biomaterials confer intrinsic molecular and mechanical properties including stiffness, swelling, and viscosity that will guide their experimental design and applications. The most frequently used in the gynecological field are natural derived polymers that include purified collagen, fibrin, gelatin or Matrigel. However, recurring limitation in maintaining multi-cellular cultures, tunability and rapid degradation have inspired the development of alternative multi-polymer or synthetic matrices. Fully or semi synthetic polymers such as PEG [65, 66], gelatin methacryloyl (GelMA) [73] or polyacrylamide offer a wide range of tunable regimes that enable the tailoring of the biophysical (e.g. stiffness), molecular (e.g. cell–integrin interactions) and dimensional (e.g. 2D, 2.5D, 3D) properties, thereby providing greater control of the cell–matrix interaction and cellular compartments. A limitation of these synthetic matrices is that although they are often designed to target and recapitulate specific ECM properties, they may not always capture the full heterogenous composition of the native tissue. Nonetheless, these hydrogel biomaterials have been shown to enable multicellular ‘organoid systems’ that can recapitulate tissue-level morphogenic processes. An alternative biomaterial strategy to maintain 3D cell cultures is the use of xenogeneic decellularized ECMs as a hypoimmunogenic tissue-derived hydrogel that offers a great potential for regenerative medicine [74] (figure 8(B)). Scaffold-free systems are also utilized as an alternative model in reproductive research as a scalable method of generating multi-cellular cultures without the immediate need of a hydrogel. These cellular aggregates (spheroids) rely on cell–cell contact to generate tissue constructs and can be generated using micro-molded agarose reactors [75], hanging drop techniques [68] and low-adhesion microwells. Altogether, these hydrogel and scaffold-free approaches highlight the variety of emerging biomaterials being used to sustain EEOs cultures, develop multicellular models in vitro and study the endometrium. Since each biomaterial confers specific physiological or technical features that will determine its applications and utility, model development must be driven by the hypothesis-driven questions being addressed.

Building upon these biomaterials, implementation of comprehensive microfluidic platforms, such as OoCs or MPS offer further opportunities to increase the complexity of *in vitro* model systems and enable the dissection of tissue-level processes. An advantage of these MPS is the ability to compartmentalize cell culture in microfluidic channels to analyze cell–cell communication and interrogate how biophysical cues influence cell behavior. For example, culturing cells in microfluidic channels enabled the study of the vascular endothelium in the endometrium and probe the role of hemodynamic forces on reproductive health [70]. Moreover, the coupling of hydrogel biomaterials (e.g. GelMA, Fibrin, PEG) with these microfluidic platforms enables the study of 3D angiogenic processes in endometrial tissues in both the gravid and non-gravid uterus [71, 73]. On-going applications of these sophisticated platforms will also help address the pressing need to incorporate and understand heterotypic immune-mediated events in the reproductive tract. Lastly, following cell–cell communication, these microfluidic models also show promise to extend these crosstalk principles to study organ–organ communication by enabling the potential to inter-connect other tissues systems (e.g. the gut, liver, ovaries) and interrogate the immune-endocrine signaling systemically with pharmacokinetic/pharmacodynamic sensitivity [72, 52]. Altogether, these approaches serve to resolve short-term (4–6 d) [64, 74] or long-term (15–28 d) [70, 72, 52]experimental needs and will help to understand the endometrial microenvironment, as well as disease pathogenesis in endometrial disorders such as endometriosis, abnormal uterine bleeding, and infertility.

The last decade has seen a growth in organoid and MPS model development for the reproductive tract, with varying levels of sophistication and complexity. Although many of these model systems remain at the prototypical stage, their impact and innovation has already helped advance our understanding of endometrial biology and offer numerous clinical implications. For example, microfluidic models have helped identify putative mechanisms to explain long-standing histological observations regarding decidualization in the human endometrium. Specifically, an OoC of the perivascular stroma was used to show how physiological cues (e.g. hemodynamic forces) impact stromal-endothelial communication and enhance stromal decidualization [70]; which may help explain why decidualization originates surrounding the vasculature. Other organoid-based heterotypic models (e.g. assembloids) also have provided tractable platforms on which to study the early establishment of human pregnancy that, when coupled to high content analysis (e.g. single cell transcriptomics), have helped identify shifts in stromal/decidual sub-populations that regulate blastocyst implantation [64]. Finally, these models have helped shape the clinical field by providing platforms to understand endometrial disorders. For example, spheroid models in collagen hydrogels have helped quantitatively dissect ECM–cell interactions in endometriosis and helped reveal the invasive behavior of endometriotic cell populations while simultaneously serving as a screening platform for putative drug therapies [68]. As these models become more sophisticated, reproducible, and readily accessible to the community, we should expect to see further novel insights, a more thorough understanding of fundamental endometrial biology and the identification of therapeutic targets for reproductive diseases.

### Concluding remarks

In summary, these advances offer to revolutionize the gynecological field by enabling new research directions and provide exciting opportunities to interrogate the fundamental biology of both physiologic and disease conditions. The development of organoids, multi-cellular systems, synthetic matrices, and microfluidics demonstrate a particularly strong potential as valuable mechanistic and phenotypic screening tools. It is important to recognize the technical and conceptual limitations to every model system and that their utility must be strategized to address specific applications. Thus, these emerging technologies must be complemented with robust validation using quantitative analysis and *in vivo* benchmarking whenever possible. There are further obstacles ahead that will need to be addressed including the reproducibility and scalability, defining media parameters for multi-cellular cultures, and biocompatibility issues of materials (e.g. polydimethylsiloxane-based models). Nonetheless, the road ahead promises a bright future for the field of reproductive health as more innovative and sophisticated biomaterials and platforms are currently being developed to address pressing biological and clinical needs.

### Acknowledgments

This work was supported in part from the Bill and Melinda Gates Foundation (INV-001780). Figures were created with BioRender.com.

## 6. Biomaterials interventions for pelvic organ prolapse (POP)

Katrina M Knight

Department of Bioengineering, University of Pittsburgh, Pittsburgh, PA, United States of America

### Status

**Figure 9.**
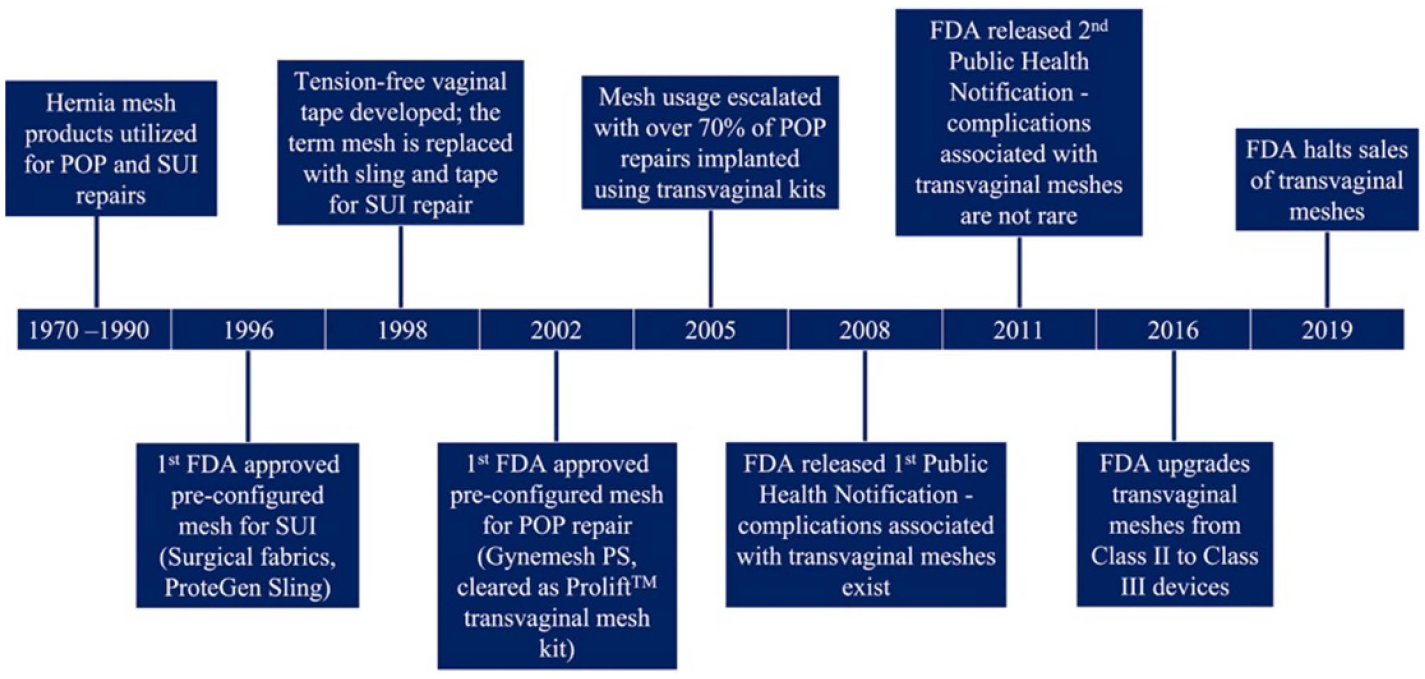
Synthetic meshes have been utilized in the treatment of pelvic floor disorders for many years. Pictured is a timeline of synthetic mesh usage in POP and stress urinary incontinence (SUI) repairs, focusing mainly on the progression of synthetic mesh in POP repairs. Reprinted from [84], Copyright (2021), with permission from Elsevier.

**Figure 10.**
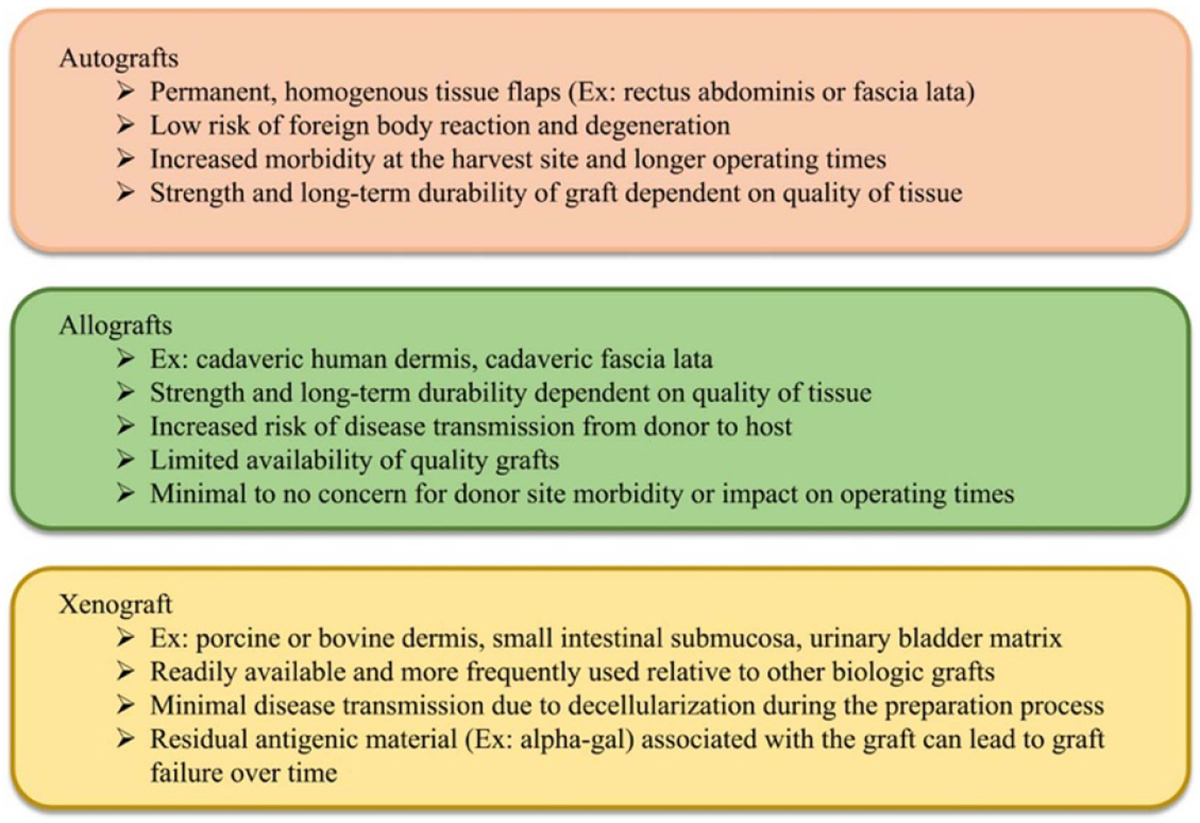
There are three main types of biologic grafts utilized in POP repairs: autografts (derived from patient), allografts (human source excluding patient), and xenografts (animal source). Examples of each type of graft as well as the associated pros and cons are discussed. Reprinted from [84], Copyright (2021), with permission from Elsevier.

POP is characterized by the unnatural descent of the pelvic organs (bladder, urethra, uterus, rectum) into the vagina. Parity (defined as the number of vaginal deliveries), advanced age, and obesity are leading risk factors for POP development [76]. Beginning as early as the mid-4th century BC, various objects such as fruits (e.g. pomegranates) and metals were used to relieve the symptoms of POP [77]. These early materials blocked the pelvic organs from protruding into the vagina, like modern-day pessaries. Current pessaries, however, are primarily manufactured from plastic (e.g. silicone) and are used to treat patients who are unfit or decline surgery and for those seeking temporary relief of POP-related symptoms. Alternatively, synthetic mesh and biologic grafts are utilized in the surgical repairs of POP (figures 9 and 10).

Surgical repairs of POP using a patient’s own tissues are associated with unacceptably high complication rates (up to 70% at 5 years [78, 79]); thus, synthetic meshes are used to re-enforce repairs. Synthetic meshes are implanted via a transabdominal or transvaginal approach. Initially FDA approved via the 510(k) mechanism, synthetic meshes are hernia mesh products marketed for POP repairs. They were not designed for the unique biologic and mechanical environment of the female pelvis. Hence, mesh usage has been plagued by complications, particularly for transvaginally implanted meshes (~20%) [80] which ultimately caused the FDA to halt the distribution of transvaginal mesh products in 2019. Though less common, there are a concerning number of complications (~10%) [81] associated with transabdominally implanted meshes. Pain and exposure of mesh fibers through the vaginal epithelium are the two most reported complications.

Once integrated into pelvic tissues, mesh cannot be easily removed (if at all) and in some instances the reoperation results in significant removal of portions of the vagina. Despite mesh removal, often complications (especially pain) fail to resolve [82]. This is particularly concerning given that approximately 12.6% of women will undergo surgery to repair POP by age 80 and this percentage is expected to rise (~50%) by 2050 [79, 83]. Ultimately, synthetic mesh complications have left limited treatment options for surgeons and their patients and has sparked a renewed interest in biologic grafts. The etiology of synthetic mesh complications is unclear and understanding the pathogenesis of mesh complications is a critical next step towards improving patient outcomes and developing alternative biomaterials to the benefit of women worldwide.

### Current and future challenges

Understanding the mechanisms of mesh complications is challenging given the ethical dilemmas with the procurement of human vaginal tissue. Research utilizing *ex vivo* mechanical testing demonstrates that mesh tensioning results in pore collapse for most meshes, which potentially leads to inadequate tissue incorporation, encapsulation, and pain [85]. Tensioning mesh also causes the mesh to wrinkle, resulting in regional increases in the concentration of mesh and a heightened foreign body response [86]. These architectural changes are observed clinically as meshes removed for complications often demonstrate pore collapse and wrinkling. Furthermore, analyzing mesh-explants removed for exposure implicate tissue degradation as a plausible mechanism of exposure whereas fibrosis has been linked to pain [87–89]. There likely exists a continuum along which mesh complications exist and understanding the factors that lead to exposure versus pain is crucial to preventing mesh complications and improving patient outcomes.

Advancements in biomaterials inventions for POP repairs are hindered by our limited knowledge of pelvic anatomy. Support to the vagina (and indirectly the pelvic organs) is provided by a complex network of connective tissues and muscles. The morphological properties (e.g. position, orientation, and shape) of vaginal anatomy consistent with a well-supported pelvis in the absence of POP is poorly defined. Furthermore, it is unclear how vaginal morphologic properties change with aging (from adolescent to adult), pregnancy and delivery, and following injury, nor how these changes are associated with POP development. Collectively, these gaps in knowledge present a challenge in developing novel biomaterials interventions for POP repairs. This is particularly true for biologic grafts and regenerative medicine approaches to POP repairs, in which an understanding of the patient specific cause of POP and the tissues needed to regenerate is key.

Identifying the ideal biomaterial for POP repairs is also a challenge. Synthetic meshes are manufactured primarily from polypropylene, which is significantly stiffer than vaginal tissue. Generally, devices that are substantially stiffer than the tissues they are designed to augment are associated with poor outcomes—a phenomenon known as stress shielding. Indeed, implanting stiff mesh onto the vagina of nonhuman primates via a transabdominal approach resulted in compromised vaginal structure and function consistent with stress shielding [90, 91]. Stiffness also was attributed to tissue degradation and mesh exposure in an ovine transvaginal model [92]. Consequently, researchers are exploring alternative (softer) materials; however, the target material stiffness is unknown. Numerous research studies have characterized the mechanical properties of the vagina and how these properties change with aging, parity, POP, and menopausal status [93]. The variations in testing protocols and inconsistences in anatomic location of samples analyzed however makes comparing results and the development of a consensus regarding the mechanical properties of the vagina difficult. Standardized protocols, increased sample sizes, and consistency in sampling location are therefore needed to manufacture a device(s) from a material that complements rather than inhibits the function of the vagina.

### Advances in science and technology to meet challenges

The development of suitable animal models is key for the advancement of basic science research as it relates to mesh complication pathogenesis. Recently, Knight *et al* reproduced the mesh complication of exposure by implanting deformed mesh (pores collapsed and wrinkled) onto the vagina of nonhuman primates [94]. Specifically, mesh deformation led to two differing responses: tissue degradation (likely mechanism of mesh exposure) and myofibroblast proliferation (likely mechanism of pain). Knowing that mesh deformation leads to mesh complications, researchers can use this approach to induce complications and fill the gaps in knowledge regarding mesh complication pathogenesis on both the tissue and cellular level. Furthermore, it will inform the design of mesh modifications and aid in the development of novel devices.

Defining what is ‘normal anatomy’ will require an interdisciplinary approach with a team of researchers and clinicians working collaboratively to assess positional changes of the pelvic organs throughout a woman’s lifespan. Recently, researchers within the field have begun to combine MRI imaging and statistical shape modeling (SSM) to assess differences in anatomy, for example differences with age, pregnancy, and POP status [95]. Relatively new to the urogynecology field, SSM will serve as a powerful tool that will enhance our knowledge of anatomic changes and normality; it will also aid in the identification of patient-specific needs (including tailored devices) for POP repairs. Furthermore, SSM, along with computational modeling, can be used to predict patients at risk for developing POP, allowing for early intervention. Ultimately, understanding what tissues are compromised in POP can lead to the development of targeted regeneration using biologic grafts.

Although studies exist, there is a need for more studies (mechanical, histologic, and biochemical) investigating the structure and function of the vagina obtained from biopsies, cadavers, and animals. Similarly, the development of novel *in vivo* devices for vaginal mechanics are warranted. Such studies and devices will aid in the development of a device (or devices) that closely mimic the vagina. The optimization of the design of novel biomaterials for POP repair will also depend on enhancing our knowledge of the host response to mesh on both a tissue and a cellular level using both human and animal tissues.

### Concluding remarks

Great strides have been made from the initial biomaterials utilized in POP repairs to now. However, there is still more to learn. The ideal biomaterial(s) for POP repairs should be designed specifically for the unique tissues and loading conditions within the female pelvis. Ultimately, filling the gaps in knowledge of anatomy, patient-specific POP etiology, vaginal structure and function, the host (vaginal) response to biomaterials, and pathogenesis of mesh complications will be crucial to the success of future biomaterial interventions. Such gaps can be filled with an interdisciplinary research approach that combines basic science (animal models, computational modeling, imaging, SSM, *in vivo* measurement and imaging techniques, etc) and clinical research. Over the next 10–15 years, it is anticipated that novel materials and techniques to improve the integration of biomaterials with the host will be developed to improve patient outcomes while concomitantly providing more options for patients.

### Acknowledgment

K M K would like to acknowledge support from the National Institutes of Health under Grant No. K12HD043441.

## 7. Biomimetic materials for placenta research

Emily M Slaby and Jessica D Weaver

School of Biological and Health Systems Engineering, Arizona State University, AZ, United States of America

### Status

**Figure 11.**
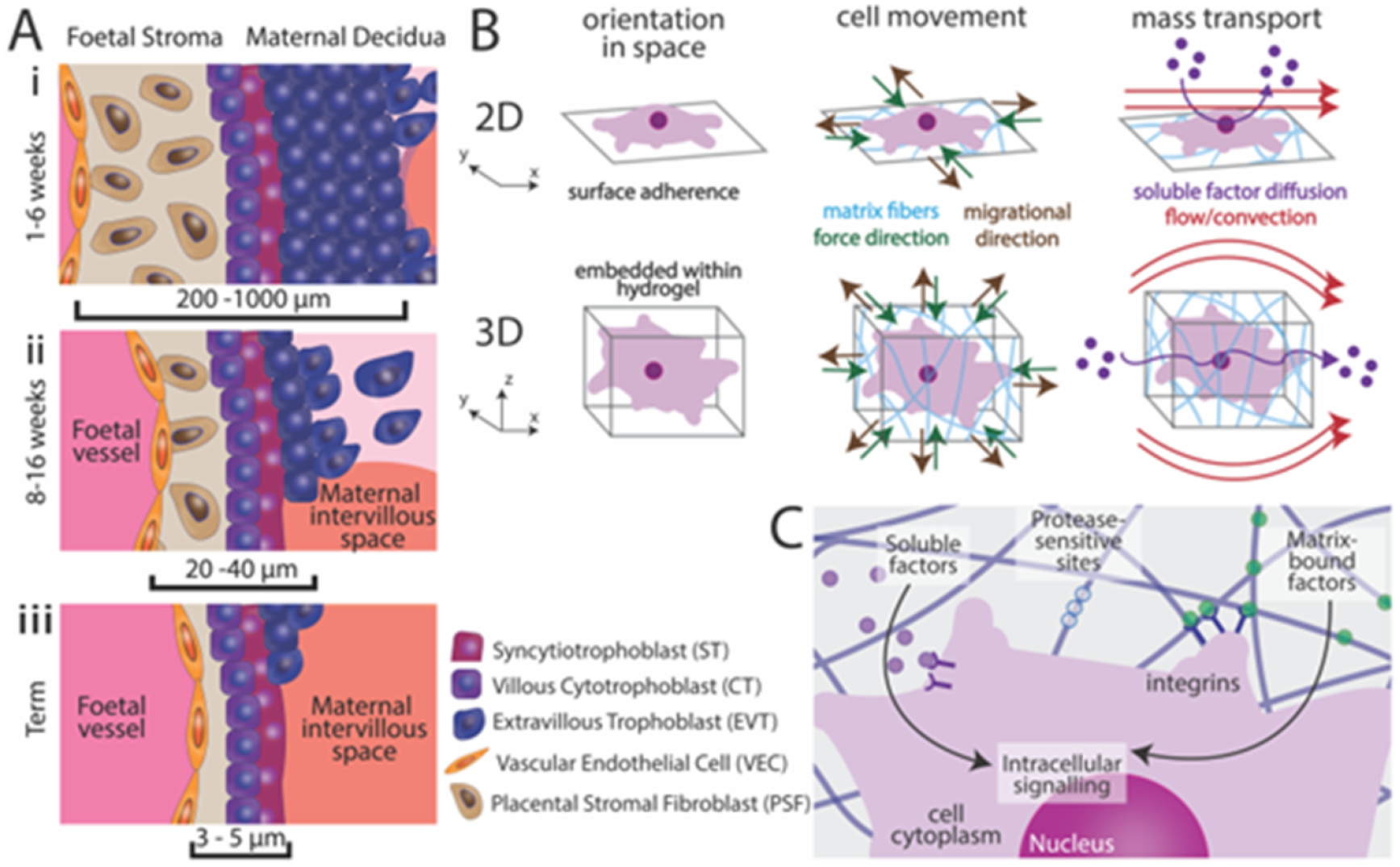
Biomimetic hydrogel systems to recapitulate placenta *in vitro*. (A) The placenta develops and changes rapidly throughout early (i), mid (ii), and late (iii) gestation, changing in cellular architecture and ECM composition to meet the nutrient needs of the developing fetus. (B) Two-and three-dimensional culture systems provide differing cues to placental cells *in vitro*. (C) A 3D biomimetic culture systems can provide cues that recapitulate the native placenta microenvironment.

The placenta is a dynamic, complex, and temporary organ that develops rapidly throughout pregnancy (figure 11(A)), continuously adapting to provide nutrients to the developing fetus and protect it from immunological attack. Placenta research is vital to understanding pregnancy complications and the mechanisms underlying successful pregnancies. The human placenta is challenging to study pre-parturition due to potential risks to the fetus, and substantial differences in placental physiology and development between species make animal models of limited use to investigate human placental dynamics. The vast majority of *in vitro* research with placental cells (e.g. cell lines or primary placenta cell isolates) or villous explants takes place in 2D culture systems, which fail to replicate the complexity and architecture of a multicellular, 3D tissue (figure 11(B)). Next-generation *in vitro* culture systems that replicate the placental microenvironment will be pivotal to advancing our knowledge of human placental physiology and immunology.

Biomaterials have been used for decades to generate artificial 3D cellular environments *in vitro* for a wide variety of tissues, though they have only permeated placenta research within the last decade. Biomimetic materials, biomaterials engineered to mimic a tissue microenvironment, can be designed to exploit and manipulate material properties such as mechanical stiffness [73, 96], ECM signaling [97–101], and soluble or matrix-tethered signaling gradients [102, 103] (figure 11(C)). Several 2D *in vitro* placenta culture systems have used surface modification to generate biomimetic synthetic materials using substrates such as polyacrylamide [97, 101], polydimethylsiloxane [98, 102], and polycarbonate [99, 100], coated with ECM components such as collagen, fibronectin, and decellularized human placenta. While these 2D systems can approximate late gestation placental transport in on-a-chip platforms (figure 11(A-iii)), they are poor models for early gestation transport, placenta remodeling and development, and immunomodulation (figure 11(A-i)); therefore, 3D biomimetic culture platforms are necessary to fully replicate the placenta microenvironment through all stages of gestation.

Within the past decade, commercially available 3D culture platforms such as Matrigel [104] and Geltrex [105] have been used to generate biomimetic culture environments to study placenta formation and cell–cell interactions. Recently, researchers have used synthetic biomimetic 3D culture systems such as GelMA hydrogels [73, 103, 106, 107] to study invasion and cell signaling in response to altered matrix stiffness. These recent advancements in 3D *in vitro* placenta culture systems demonstrate promise in generating physiologically relevant biomimetic materials with a high potential for impact in placenta research.

### Current and future challenges

With a dearth of healthy placental cell and villous explant samples available from early and mid-gestation pregnancies, the development of 3D biomimetic *in vitro* culture models is critical to advancing early gestational stage human placenta research. Non-primary tissue cell sources for these models include trophoblast cell lines, established trophoblast progenitor stem cell lines, and induced pluripotent stem cell-derived trophoblasts. The vast majority of research on these cells has been conducted on 2D surfaces [97, 101, 102] or in commercially available 3D matrices [104, 105] like Matrigel, which are poor replicas of the native placenta microenvironment. Matrigel, which is derived from decellularized murine tumors, provides a rich environment of ECM and soluble factors to encapsulated cells; however, the substantial batch-to-batch variability in these platforms leads to poor experiment reproducibility, which is a common challenge with naturally-derived materials. While 2D systems like placenta-on-a-chip [98–100] models are effective for studying the transport of drugs and molecules across the placenta, these systems primarily replicate late gestation transport and fail to reproduce the complexity of early and mid-gestation placenta architecture (figure 11(A)).

Recapitulation of the multi-cellular placenta architecture *in vitro* has remained elusive, and organoid generating techniques often generate an inverted structure. Future challenges in human placenta research necessitate the development and application of biomaterial systems with precise control over biomimetic signaling that mimic the dynamic changes and signaling of the fetal–maternal interface (figure 11(C)). Through the course of gestation, the placenta exhibits gradients of ECM composition, nutrients, metabolites, and hormones that drive the development and infiltration of the maternal decidua. Simulating these gradients in synthetic culture systems will be critical to accurately model the placenta. Additionally, biomimetic systems will require control over matrix stiffness and porosity and should facilitate cell-responsive remodeling of the matrix. Finally, the chemistry used to fabricate such a matrix must be minimally cytotoxic to avoid altering the encapsulated cell phenotype. Surmounting these simultaneous challenges would yield effective biomimetic material platforms for placenta research.

### Advances in science and technology to meet challenges

To advance human placenta research, rationally designed and dynamic biomimetic material platforms are necessary to replicate the early and mid-gestational placenta microenvironment. Current placenta-on-a-chip designs [98–100] have generated increasingly complex models of late gestation placenta with biomimetic materials and multiple cell types; however, *in vitro* models of early- to mid-gestation placenta will need to exploit 3D biomimetic platforms to replicate the complex multi-cellular placenta architecture. Future biomimetic material platforms must recapitulate placenta mechanical stiffness, ECM composition, dynamic remodeling, and soluble and immobilized signaling gradients to approximate normal and disordered placenta microenvironment states. For example, modifying ECM composition and matrix stiffness could enable the mechanistic study of disorders of placental development and implantation [97, 101], such as placenta accreta, increta, and percreta. Similarly, modulation of local nutrients and soluble and immobilized signaling molecules can facilitate studies of placental mechanisms of immune tolerance and associated disorders, such as preeclampsia.

Rapid biomaterial advancements over the past few decades have resulted in a plethora of biomimetic material platforms. Naturally-derived matrices such as decellularized ECM-based hydrogels can be used to provide matrix signaling to embedded cells, though mechanical stiffness is challenging to control in these matrices. Conversely, synthetic polymer-based hydrogels offer a high degree of control over matrix mechanical properties, but they must be modified to imbue biomimetic signaling. Additionally, synthetic hydrogels have less batch-to-batch variability than natural matrices, which could result in more reproducible experimental outcomes. The available range of biomimetic material platforms continues to grow, with an increasing capacity to control spatiotemporal factors within matrices. Expanded libraries of cytocompatible, biomimetic platforms will be necessary to accelerate studies of normal and disordered placenta development in the next decade.

### Concluding remarks

Biomimetic materials are a powerful emerging tool in the field of placenta research, enabling the generation of 3D culture systems that better recapitulate the native placenta microenvironment. Advanced biomaterial-based *in vitro* culture systems may enable the study of human placenta development, physiology, and immunology in an unprecedented manner, and expanding this area of research could have broad impacts on maternal mortality, diseases of pregnancy, and understanding of immunological tolerance mechanisms.

### Acknowledgments

The authors are supported by the Juvenile Diabetes Research Foundation Innovator Award (1-INO-2020–915A-N), the Arizona Biomedical Research Centre New Investigator Award, and the National Institutes of Health Director’s New Innovator Award (DP2AI169476).

## 8. Biomaterials for microfluidic placental function assays

Nicole N Hashemi

Department of Mechanical Engineering, Iowa State University, Ames, IA, United States of America

### Status

The human placenta is a critical and complex organ that performs various functions. These features include several of the following: safe circulation and flow of fetal and maternal blood, nutrients, and oxygen; elimination of waste and carbon dioxide; and protection from diseases, infections, and xenobiotics from mother to fetus. Performing these actions supports fetal development and maturation [108–110]. The placenta is composed of the syncytiotrophoblasts and the fetal capillary endothelial layer. The placental barrier separates the fetal circulation from the maternal blood so that the two bloodstreams do not mix [99]. Placental examination and monitoring are complicated given the considerable changes in placental structure during pregnancy [111]. Needless to say, as pregnancy progresses, the mother’s placental barrier changes more dramatically, making the monitoring process of the fetus and placenta itself even more difficult [111]. Although placenta research and monitoring are complex, the knowledge already gained from the placenta has proven to be an essential source of information to understand the overall performance of the human placenta. Therefore, placental physiological function and structure play an important role in investigating, monitoring, and understanding the characteristics of the human placental barrier. Understanding placenta physiology lays the foundation for researchers to assess the human placenta and create models for the scientists and pharmaceutical industries to understand it better. However, conducting such studies is time-consuming and could pose a risk to the fetus in *in-vivo. Ex-vivo* studies are limited regarding the reliable assessment of transport mechanisms in the human body. Placenta-on-a-chip models provide opportunities to test and understand the effects of medicines on the placenta while eliminating the risk of fetal and maternal rupture and destruction [99, 112, 113].

### Current and future challenges

**Figure 12.**
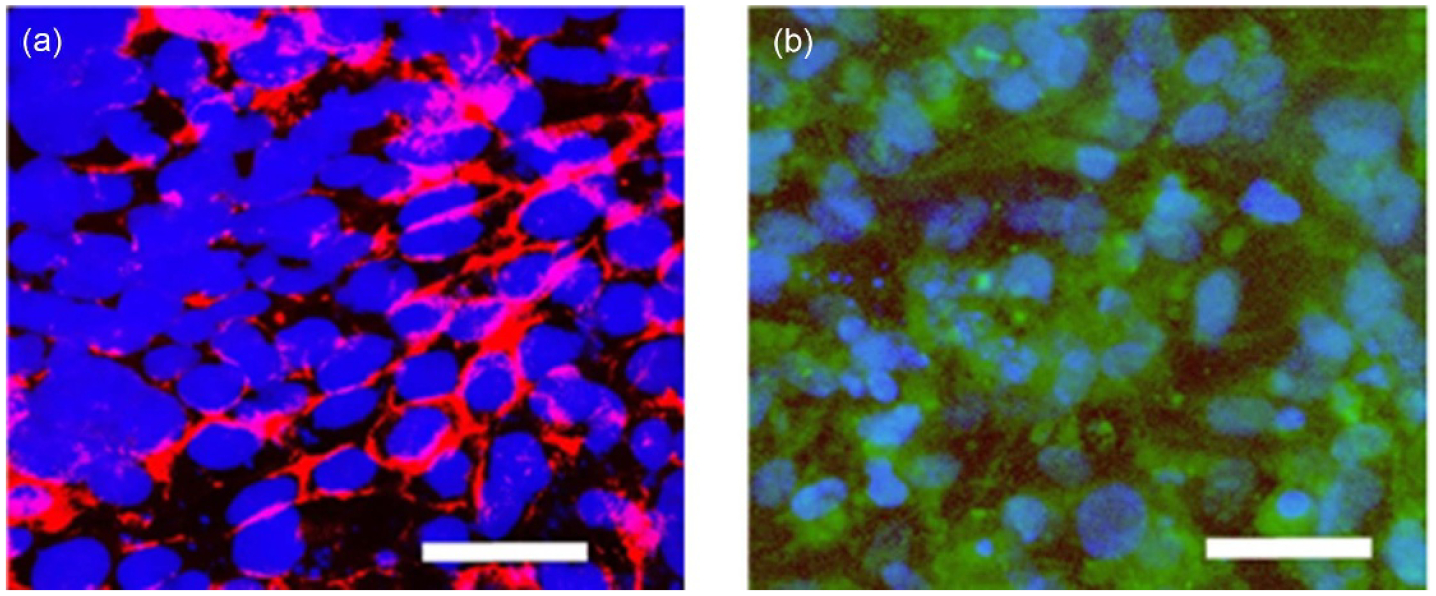
Cells in channels after 48 h of media perfusion. (a) BeWo cells show epithelial adherence junctions with E-Cadherin and Nuclei labeled with DAPI staining, (b) HUVECs show endothelial adherence junctions with VE-Cadherin and Nuclei labeled with DAPI staining. Scale: 50 *μ*m.

The placenta-on-chip has made significant strides in testing drugs across microfabricated placental barriers, but this model has only recently been developed compared to *in-vivo* and *ex-vivo* models. One of the main challenges in the placenta-on-a-chip models is to make sure that the models can perform under a few efficient drug intake tests; this is partly owing to the fact that researchers have devoted most of their time in recent years to modifying the procedure for producing placental chips. Scientists have devoted a significant amount of work to improve the fabrication and operation of the placenta-on-a-chip; yet, researchers are unable to replicate the crucial physiological properties of the placental barrier. For instance, placental chips lack assays for lipids and amino acid transport through the placental barrier, which are crucial qualities for sustaining chip performance and placenta responsiveness and replicating the human placenta’s physiology [99]. In contrast to *in-vivo* and *ex-vivo* models, the *in-vitro* technique has been tested more thoroughly throughout model development than earlier models. There are concerns regarding the expertise of pharmaceutical study models as well as the capability of the chip to undertake complex tests and monitor polydimethylsiloxane chips. For instance, as the functionality and complexity of the 3D co-cultured cell model rises, microarray analysis for high-resolution tissue screening and visualization of tissue location becomes more challenging to conduct [114]. In the biological response assay, the limited number of cells on the chip compared to the *in vivo* placenta impedes the detection sensitivity of drug uptake and cellular interactions. As a result, the presentation and assessment of the chip’s clinical results make it difficult for pharmacists to communicate data to patients [115]. Moreover, it has been reported that due to the size of cell culture chambers and channels, the shear stress developed is significantly lower than the shear stress developed in fetal capillaries [108]. In addition, further studies are required to assess the effect of shear stress on the formation of syncytiotrophoblasts since It is possible for trophoblasts to detect flow shear stress via mechanotransduction; blood flow shear stress rates through invading uterine spirals range from 1 to 10 dyn cm^−2^; however, the shear stress lowers to less than 0.1 dyn cm^−2^ in the intervillous area, according to studies [116]. The cells subjected to shear stress after differentiation into trophoblast-like cells showed proof of the formation of syncytium compared to those not subjected to shear stress when trophectoderm-like cells from human naive induced pluripotent stem cells were placed under a shear stress of 10 dyn cm^−2^. Consequently, shear stress has been found to be essential for the syncytialization of trophoblasts [116]. Additionally, multiple cell lines of the human placenta should be used to better mimic the physiology and anatomy of the human placenta developed for placenta-on-a-chip studies [108]. Figure 12 shows an *in vivo*-like microsystem with HUVECs representing the endothelium, trophoblasts (BeWo) cells representing the epithelium, and a semipermeable membrane representing the placental barrier.

### Advances in science and technology to meet challenges

As *in-vitro* approaches continue to be developed, the capability to perform a variety of investigation on micro-engineered barriers for pharmaceutical trials are realized. However, more disease-related studies are required to advance the mimicking of the placental barriers. Also, by using these *in-vitro* models, scientists may gain new insights into the developmental stages of foetation. For example, one will gain knowledge of placental development and how the transport of compounds affects fetal growth. However, the applicability of the universal model has been thoroughly discussed, and outlined that the model must simulate the physiological properties of the human placenta *in-vivo* while ensuring that procedures and methods are performed in the same way for all. Developing a working model for conducting drug intake studies is vital for a better assessment of the influences of drugs on the maternal–fetal barriers. Moreover, including genetic studies to assess the placental expression of genes could help researchers in examining their models to achieve a model which is closer to the actual *in-vivo* studies [116]. Creating truly representative placenta models that accurately mimic the human placenta’s significant changes during different pregnancy stages remains a challenge. This has inspired the scientific community to exploit technological advances to overcome these obstacles. In addition, maintaining the confluency and functionality of trophoblasts for a prolonged time are critical challenges to address. The continued funding and support for the placenta-on-a-chip models will aid scientists in advancing the device’s overall functionality. Scientists will be able to conduct various experiments, other than pharmaceutical and drug testing, across the micro-engineered barrier in the future as researchers continue to innovate on the *in vitro* technique.

### Concluding remarks

The application and further development of placenta-on-a-chip for pharmacokinetic/pharmacodynamic studies of the human placenta will aid the reduction of drug-induced congenital disabilities and a better understanding of the effects of these drugs on the human placenta. The significance of the placental barrier during pregnancy has been investigated for decades, but the newly emerged techniques have enabled new *in-vitro* technology that accurately mimics the anatomical structure and physiological function of the placenta and the barrier. Multiple placenta-on-a-chip [99, 108, 116] platforms have appeared from the imitation of both placental barrier anatomy and physiology and deepens the understanding of this vital organ system. With the advent of more advanced placenta-on-a-chip models of the placental barrier, such models are expected to substantially change how research and development are done in this field.

### Acknowledgments

This work was partially supported by the Office of Naval Research Grant N000141712620 and National Science Foundation Award 2014346.

## 9. Biomedical engineering for cervical insufficiency in pregnancy

*Yali Zhang*^1^ *and Michael D House*^1,2^

^1^ Mother Infant Research Institute, Tufts Medical Center, Boston, MA, United States of America

^2^ Department of Obstetrics and Gynecology, Division of Maternal Fetal Medicine, Tufts Medical Center, United States of America

### Status

**Figure 13.**
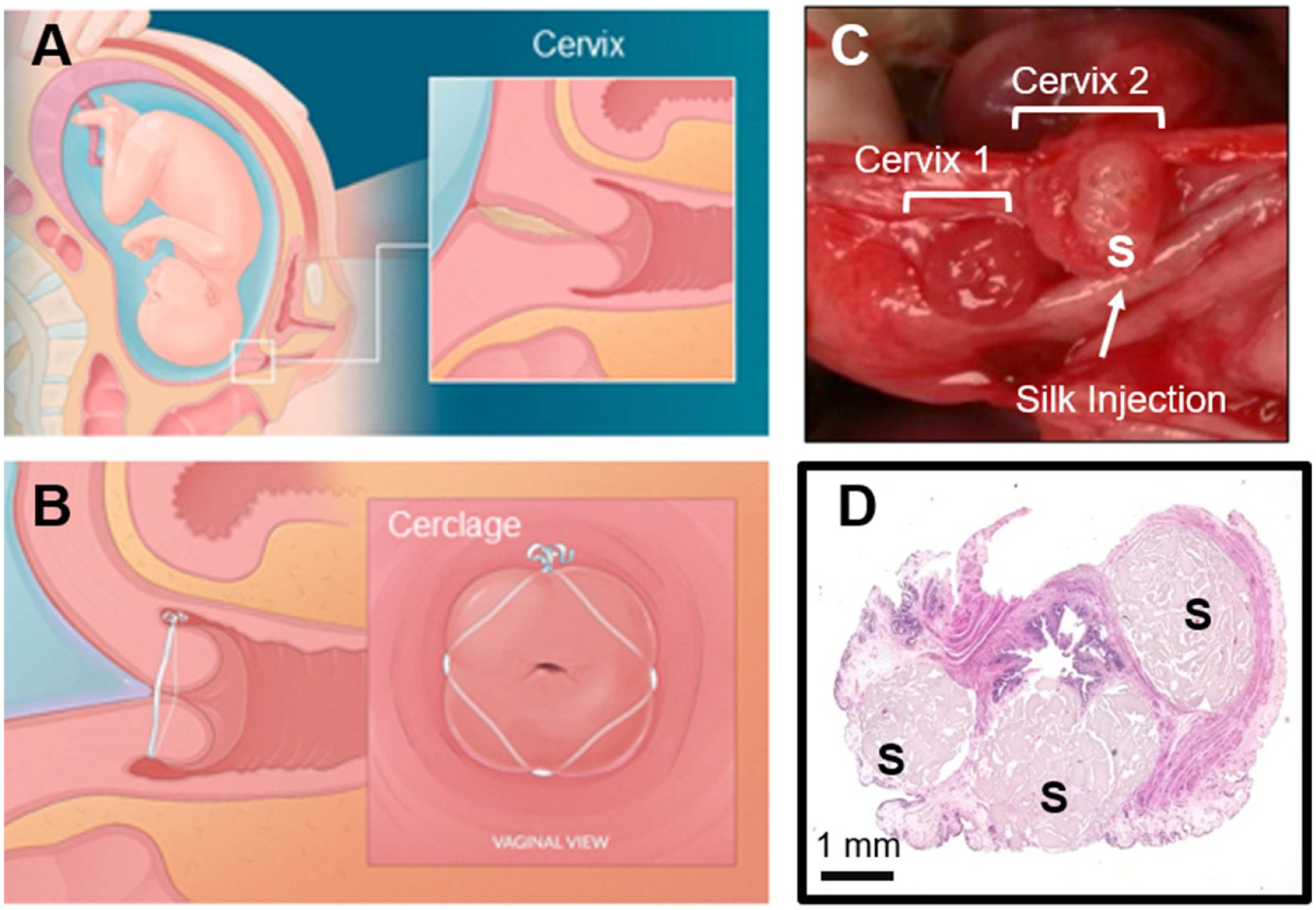
(A) Sagittal anatomy of the cervix during pregnancy. (B) Anatomic position of a cerclage. (C) The rabbit has two separate cervices. In this experiment, one cervix was injected with silk hydrogel and other cervix served a control. (D) Histology cross section showing the silk injections in the cervical stroma. S, silk hydrogel.

Cervical insufficiency is a well-known complication of pregnancy that leads to preterm birth. The cervix forms the lower portion of the uterus (figure 13(A)). Normally, the cervix remains closed as the fetus grows. At term, the cervix dilates under the influence of uterine contractions. Cervical insufficiency describes a clinical condition in which the cervix dilates prematurely. In cervical insufficiency, the cervix dilates as the fetus grows, which can lead to a pre-viable or a peri-viable birth. Deliveries prior to viability are a miscarriage. Infants born in the peri-viable period can suffer long-term health consequences such as chronic lung disease, blindness and cerebral palsy.

The static function of the cervix is critically important for a full-term pregnancy [117]. During fetal growth, multiple static stresses act to dilate the cervix. Static stresses arise from uterine distension, the hydrostatic pressure of the amniotic cavity, and anatomic variations of the pelvic structures. Countering these static stresses are the strength of the fibrous connective tissue of the cervical stroma, and the adhesion of the fetal membranes on the cervix [118]. When static stresses exceed cervical strength, the cervix shortens and dilates, which can lead to cervical insufficiency.

The current standard of care treatment for cervical insufficiency is cerclage suture (figure 13(B)) [119]. Under regional anesthesia, a non-absorbable suture is placed around the cervix, with 4–6 passes of the needle through cervical tissue. After traveling around the cervix in a purse string fashion, a knot is tied. A cerclage suture is placed in the first or second trimester of pregnancy, and it is removed near the due date. The most common type of suture used for cerclage is polyester braided tape (Mersilene, Ethicon RS23). The rationale of cerclage suture is to support the cervix and prevent preterm dilation. The cerclage is removed when there are signs of labor or at 37 weeks of gestational age.

This section will discuss our recent efforts to use TE to study the connective tissue of the cervical stroma and to explore a novel injectable hydrogel to treat cervical insufficiency.

### Current and future challenges

The cervical stroma is the load bearing tissue of the cervix and impaired load bearing properties could lead to cervical insufficiency. The cervical stroma is primarily composed of collagen (dry weight 80%–90%) in humans [120]. During pregnancy, the stroma softens significantly in preparation for childbirth. Mechanical testing of human cervical tissue *in vitro* demonstrated peak stresses in compression are an order of magnitude less for cervical tissue from pregnancy compared with non-pregnant tissue [121]. Aspiration testing of human cervical tissue during pregnancy confirmed significant softening as pregnancy advances [122]. Although cervical softening is readily measured both *in vitro* and *in vivo*, the biochemical mechanisms that cause cervical softening are incompletely understood.

Animal models have shed light on how changes in cervical ECM lead to cervical softening [123, 124]. Cervical softening is presumed to arise from changes in the fibrous collagen of the cervix. Studies of pregnant mice demonstrated significant decreases in collagen cross-link density during pregnancy, which correlates with increased collagen solubility and decreased collagen organization on transmission electron microscopy [125, 126]. Changes in collagen cross-links, increased collagen solubility and decreased collagen organization is also seen in biopsy studies of the human cervix [120, 127].

Model systems for investigating cervical softening have important limitations. Studies of the cervix in animals may not reflect cervical changes in humans [124]. Studies of the human cervix *in vivo* are limited by the difficulty of obtaining biopsy samples. Challenges of traditional model systems have motivated our efforts to develop an engineered model of human cervical tissue (discussed below).

In addition to studying engineered cervical tissue, we are investigating an alternate treatment for cervical insufficiency. The cerclage procedure was originally described by Drs Shirodkar and McDonald in the 1950s and has remained unchanged since that time. There have been few attempts to reconsider treatment for cervical insufficiency with the goal of improving efficacy and decreasing adverse effects. In terms of efficacy, the largest randomized trial of cerclage placed in the first trimester (*n* = 1292 patients) showed a small benefit of cerclage. Delivery prior to 33 weeks occurred less frequently in the cerclage group (13%) compared with the control group (17%, *p* = 0.03) [128]. For second trimester patients with a short cervix, cerclage placement showed clinical benefit but was still associated with a 15% risk of delivery prior to 28 weeks [129]. In addition, adverse effects can be seen with cerclage suture such as cervical laceration, which occurs in 4.8%–7.9% of cases [130, 131].

A significant challenge of improving cerclage treatment is that the biomechanical environment of the cervix during pregnancy is understudied. Only in recent years have groups attempted to work out the 3D stress state of the cervix during pregnancy [118, 132]. An improved understanding of cervical biomechanics is critically needed to understand the mechanism of cerclage efficacy and rationally design improvements for cervical insufficiency.

### Advances in science and technology to meet challenges

To address the limitations of current models of cervical softening during pregnancy, engineered models of cervical tissue are being investigated [133]. During pregnancy, it is known that cervical tissue is exposed to multiple factors that could decrease cervical stiffness including changes in hormone concentrations, inflammatory conditions, and mechanical loading. To study cervical tissue without the complexity of the environment *in vivo*, an engineered cervix was developed. Human cervical FBs were isolated from explants of non-pregnant cervical tissue. FBs were seeded on porous silk scaffolds and cultured in spinner flasks. After 4 weeks, the seeded scaffolds produced an ECM with biochemical components and histological appearance similar to native cervical stroma. Using the engineered cervix model, the influence of steroid hormones on ECM properties was studied [133]. The engineered cervix model suggested estradiol promoted ECM growth and progesterone had a softening effect. We expect the 3D engineered cervix model will permit studies not possible with animal models or human biopsy studies.

To study a potential alternative to cerclage surgery, injectable hydrogels for cervical augmentation are being investigated (figures 13(C) and (D)) [134]. Cerclage requires regional anesthesia for placement. Also, cerclage removal can be challenging, especially if labor is present. An injectable treatment is a non-surgical approach that avoids surgical risks and avoids the need to remove the cerclage. An injectable hydrogel creates a composite tissue which could have improved properties (e.g. increased stiffness) for better function. Also, in contrast to a cerclage, injectable hydrogels increase cervical volume, which could improve barrier properties between the vaginal and uterine environments. In a recent study, purified silk fibroin protein was crosslinked with horseradish peroxidase and hydrogen peroxide to create an elastic hydrogel with mechanical properties similar to human cervical tissue [134]. In a rabbit model of pregnancy, the silk-based hydrogel was injected into the cervix. The hydrogel demonstrated cervical augmentation, biocompatibility, and biodegradation. Compared to controls, no adverse effect was seen in terms of kit delivery and kit viability. Future studies will compare an injectable treatment to cerclage in terms of improved barrier properties and mechanical support.

### Concluding remarks

Biomedical engineering offers rigorous and innovative strategies to study cervical function in pregnancy. Patients with cervical insufficiency suffer structural failure of the cervix. Engineers have the tools and training to assess structural failure and improve structural support. In addition, TE strategies are yielding new insights into cell–matrix interactions of cervical tissue. These studies show the considerable potential for using a biomedical engineering approach for cervical insufficiency in pregnancy.

### Acknowledgments

We gratefully acknowledge support from the following NIH Grants 5R01EB021264, 5P41EB027062 and 5K12HD000849. We also acknowledge support from the Bridge Funding Award from the Society for Maternal Fetal Medicine.

## 10. Precision biomaterials as tools to determine sex-specific mechanisms of cardiovascular disease

*Brandon J Vogt*1,2 *and Brian A Aguado*1,2

^1^ Department of Bioengineering, University of California San Diego, La Jolla, CA, United States of America

^2^ Sanford Consortium for Regenerative Medicine, La Jolla, CA, United States of America

### Status

Cardiovascular disease remains the leading cause of death in the United States in both men and women [135]. Across a variety of cardiovascular diseases, men and women show sex dimorphisms in disease progression, morphology, response to treatments, and outcomes. For example, adult women with ST-segment elevation myocardial infarcts are nearly twice as likely as men to die within 30 d of receiving in-hospital care [136]. A historical failure to account for sex dimorphisms in the majority of biomedical research has likely contributed to the observed sex differences and inequities in cardiovascular disease treatment outcomes. Women have been significantly underrepresented in cardiovascular disease clinical trials, leading to results that are biased towards the clinical outcomes of men [137]. Additionally, failing to identify the sex of the cells and/or animals used in most research studies has been detrimental to understanding female pathophysiology, since most research studies use male cells and animal subjects [138]. To change course, the National Institute of Health began requiring sex as a biological variable to be considered in pre-clinical studies in 2016, with calls to integrate sex and gender more effectively throughout biomedical research and device design [139].

Biomedical devices have also been traditionally designed and engineered using male-centric approaches [140], which underscores the need for engineers to join the movement to include sex as a biological variable in research and device development. For instance, biomaterials serve as enabling tools for *in vitro* and *in vivo* disease modeling, medical devices, and TE. However, biomaterials research still lags in accounting for sex dimorphisms at all length scales [141], often leading to male-biased responses to biomaterial implants. For example, women undergoing transcatheter aortic valve replacement have a 13% higher standardized in-hospital mortality rate relative to men [142]. Sex differences in valve replacement outcomes may be due in part to differences in tissue biomechanical properties, as male carotid arteries have been shown to have a higher failure strain than female arteries [143]. Given the significant sex dimorphisms observed in cardiovascular diseases and recent advances in biomaterial design, biomaterial engineers have a unique opportunity to characterize sex-specific mechanisms of cardiovascular disease progression, potentially leading to more targeted, sex-specific treatments. Ongoing areas of biomaterials research include (a) investigating intracellular sex differences using *in vitro* cell culture platforms and (b) engineering biomaterial implants that interact appropriately with the host by accounting for sex-specific tissue heterogeneities and immune responses.

### Current and future challenges

**Figure 14.**
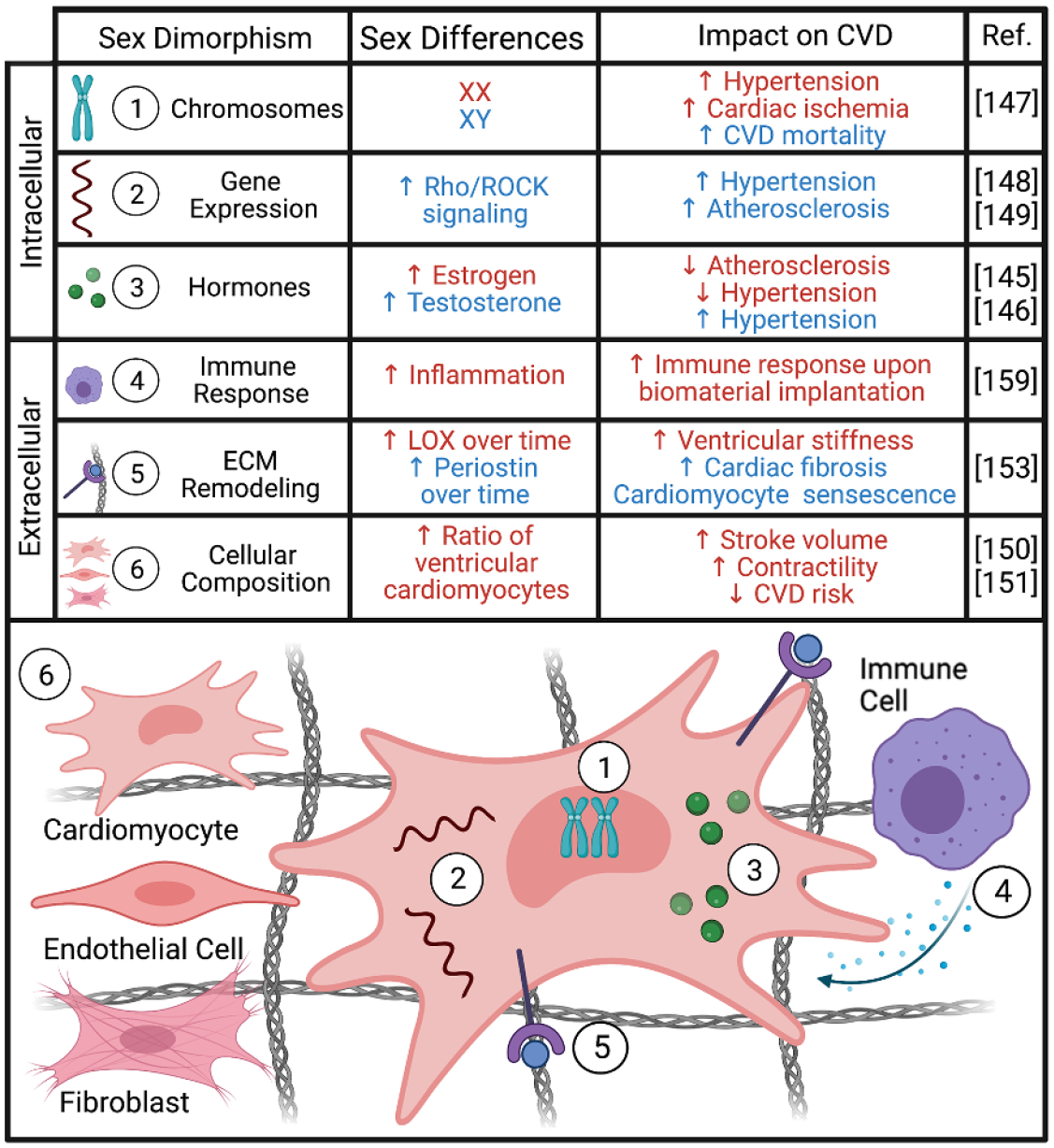
Intracellular and extracellular sex differences with corresponding effects on cardiovascular disease. Female characteristics are shown in red and male characteristics are shown in blue. Abbreviations used: CVD, cardiovascular disease; Ref., references; ROCK, Rho-associated protein kinase. Created with BioRender.com.

To create improved *in vitro* models, precision biomaterials [144] may be used to probe intracellular sex differences, including hormonal, chromosomal, and transcriptomic differences (figure 14). Hormone effects on cardiovascular disease have been well documented, with studies finding that testosterone loss in aging men and estrogen loss in women during menopause lead to increased risk of coronary artery disease and myocardial infarction [145]. Hormone effects have also been explored within the transgender community, with studies showing that transgender women receiving estrogen hormone therapy were at an increased risk for ischemic heart disease, whereas transgender men receiving testosterone hormone therapy experienced elevated hypertension risk [146]. Outside of hormone biology, the genetic and epigenetic effects of sex chromosomes on cellular phenotypes must also be considered. For example, men with rare cases of Y polysomy (two Y chromosomes) have increased cardiovascular mortality, while women with X monosomy (one X chromosome) have higher incidence of ischemic heart disease, suggesting the X and Y chromosomes play a role in cardiovascular disease mechanisms [147]. Significant sex differences have also been observed in the cardiac transcriptome, with previous studies finding that male cardiac myocytes have increased Rho/Rho-associated protein kinase signaling activity relative to female myocytes [148, 149]. Open questions remain regarding how genetic and epigenetic modifiers expressed on sex chromosomes impact how cells in culture respond to engineered microenvironments.

In addition to cell culture, precision biomaterials can also be designed as implantable devices to explore sex dimorphisms at the organism scale. For example, female human hearts have a significantly higher proportion of ventricular cardiomyocytes relative to other cells than male hearts, which increases heart contractility and stroke volume in women [150, 151]. As such, implantable engineered cardiac tissues may need to be designed to reflect heterogeneous populations of cardiac cells. Another biomaterial design challenge is accounting for sex differences in the composition of the cardiac ECM. Exploring microscopic sex differences in the ECM could offer insights into observed macroscopic sex differences in heart anatomy, such as increased left ventricle diameter and posterior wall thickness in men [152]. For example, female mice experience significant increases in lysyl oxidase, an enzyme involved in collagen fibril cross-linking, offering a possible mechanism for the increased ventricular stiffness observed in women [153]. Beyond sex differences in cellular and ECM composition, additional studies focused on how male and female immune systems respond to biomaterial implants or particle-based therapeutics are needed [154].

### Advances in science and technology to meet challenges

Simple changes can be made to improve sex-specific cardiovascular research, beginning with quality control practices such as reporting the sex of all cells, serum, and animal models used in future studies. Additionally, culturing cells in phenol red free media with charcoal stripped serum will help reduce the confounding effects of estrogens and androgens in cell culture studies. Cell culture may be further improved by optimizing sex-specific media formulations containing physiologically relevant hormone levels.

A diverse set of *in vitro* and *in vivo* tools are being developed to more accurately mimic components of cardiovascular tissues and may be leveraged to reveal sex differences in cellular behavior and tissue/organ function. For example, models of matured human cardiac tissues generated from pluripotent stem cells and fibrin gels can be used to model sex-specific responses to drugs, hormones, or other small molecules [155]. Furthermore, biomaterials with tunable stiffness show promise for exploring the synergistic effects of mechanical (e.g. tissue stiffness) and biochemical cues (e.g. inflammatory factors) on gene expression. For instance, valvular interstitial cells have been cultured on hydrogels in the presence of serum from aortic valve stenosis patients to recapitulate sex-specific phenotypes in valve disease patients, which is partially regulated by genes that escape X chromosome inactivation [156, 157]. Moreover, computational models have been developed to predict patient-specific gene expression and drug responses in valve myofibroblasts on hydrogels [158]. Additionally, tissue engineered vascular grafts are being used as implantable scaffolds to study sex differences in cellularity, inflammatory response, and biomaterial degradation [159]. Taking a different approach, other researchers have turned to using decellularized heart valves to preserve the sex-specific tissue microenvironment and explore sex differences in inflammatory responses to the ECM after implantation [160]. As biomaterials are engineered as implantable devices, an improved understanding of how material degradation impacts sex-specific, systemic inflammatory responses will be needed.

### Concluding remarks

Given that sex dimorphisms exist in nearly every aspect of the cardiovascular system in health and disease, *in vitro* and *in vivo* models that seek to understand mechanisms behind cardiovascular disease must no longer ignore sex as a biological variable. Models that recapitulate sex-specific differences apparent in human disease will become increasingly important to identify mechanisms of disease more accurately. Moving forward, precision biomaterials will serve as important tools to engineer more accurate models and help identify the sex-specific mechanisms that drive cardiovascular disease progression. As these sex-specific mechanisms are better characterized, treatments will be developed that are better targeted to the individual, with the goal of achieving equity in treatment outcomes irrespective of sex.

### Acknowledgments

B A A acknowledges funding from the NIH (R00 HL148542) and the Burroughs Wellcome Fund Postdoctoral Enrichment Program.

## 11. Sex differences in tissue repair

*John C Bradford*^1^ *and Jennifer L Robinson*^1,2^

^1^ Bioengineering Graduate Program, University of Kansas, Lawrence, KS, United States of America

^2^ Department of Chemical and Petroleum Engineering, University of Kansas, Lawrence, KS, United States of America

### Status

**Figure 15.**
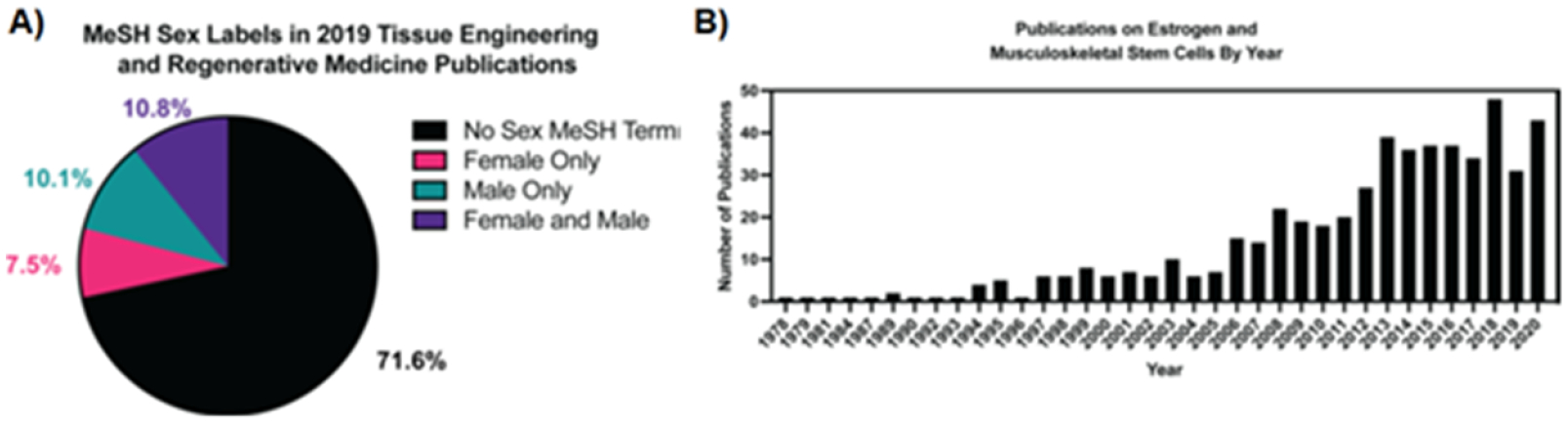
Percentage of TE and regenerative medicine publications with sex MeSH terms in 2019. (A) Publications on estrogen and musculoskeletal stem cells by year in PubMed. Search was performed on 5 March 2021 using the following search terms: estrogen AND stem cell AND (bone OR muscle OR adipose OR cartilage OR tendon OR ligament) AND (proliferation OR apoptosis OR senescence OR viability OR differentiation) NOT (cardiovascular OR cancer OR urogenital system OR haematopoiesis) (B). Reproduced from [3]. CC BY 4.0.

For functional tissue regeneration post injury, congenital defect, or tissue resection, it is critical to understand the driving mechanisms that promote regeneration rather than fibrotic scarring to reduce the onset of degenerative disease [161]. Functional tissue healing after injury follows a temporal process of controlled inflammation to clear tissue debris, cell migration to the injury site, proliferation, and new ECM production to close the wound. This process primarily involves immune cells, resident and circulating progenitor cells, and resident FB-like cells [162]. Historically, research conducted on mechanisms of tissue repair and regeneration focused on male samples and pathologies that were assumed to be directly translated to female tissue and disease. However, an individual’s biological sex significantly affects their ability to regenerate functional tissue [163]. Examples include the reduced ability for women to heal and regenerate new, healthy epidermis [164], cartilage [165], fibrocartilage [166], bone [167], skeletal muscle [168, 169], smooth muscle [168, 169], cardiac muscle [168, 169], adipose [170], and neural tissue [171] after menopause, which results from a significant loss of sex hormone signaling and disproportionately enhances the risk for many degenerative diseases including osteoporosis, osteoarthritis, cardiovascular disease, and Alzheimer’s disease. The repair and subsequent regeneration of these tissues is modulated by macrophage polarization which is directly impacted by estrogen signaling [164, 168–170]. Based on the NIH Office of Research on Women’s Health’s 2020 Strategic Plan [172], there is a push to ‘incorporate findings of sex/gender differences in the design and application of new technologies, medical devices, and therapeutic drugs’. However, in a PubMed search of TE and regenerative medicine publications from 2019, only 28.4% of the 10 651 publications reported subject sex at all [163] (figure 15(A)). Of that subset of studies, only 38% reported using both male and female samples. Such issues highlight the need for including sex as a variable in preclinical studies, specifically those focused on regenerative therapies. Also, the clinical and epidemiological data and the mechanisms that drive sex differences in tissue regeneration have lagged.

While it is exciting to see increased focus interrogating sex differences in tissue repair and regeneration such as with bone in the context of osteoporosis, this is still uncharted territory. Until there are sex-specific diagnosis considerations, treatment options, and therapy modalities for tissue regeneration in diseases with female predispositions, there is still much to do. In a recent review, we compiled the current knowledge on estrogen’s role in maintaining musculoskeletal cell stemness and reducing senescence [163]. Surprisingly, not many studies have been conducted to glean much understanding (figure 15(B)). Thus, a mechanistic understanding of what drives sex differences in tissue repair and regeneration is needed.

### Current and future challenges

The main challenges limiting advancement in strategies to account for sex differences in tissue repair and regeneration are deciphering a mechanistic understanding from both clinical and *in vitro* studies, ensuring reproducibility of the results, and the understudied mechanisms of sex differences in repair and regeneration. First, it is difficult to determine causality from epidemiological data due to confounding variables including fluctuating sex hormone levels with wide patient variability, hormone receptor levels, and environmental factors [173]. To address this, *in vitro/ex vivo* and/or animal studies are conducted to determine the sex-dependent factors responsible for the observed effect. The first area of focus is relevant models for differentiating and understanding sex hormone signaling response in biological male and female cells based on phenotype and source. Based on current knowledge, there are many unknowns about the receptors involved, receptor-mediated signaling, sensitivity of the receptor based on source, and resulting signaling pathway involved in transcriptional effects. Second, how these parameters change based on the cell host source and the cell–material interactions based on differences in structural and mechanical cues of the cell microenvironment have not been investigated. Finally, to understand the biochemical, structural, and mechanical pathways involved in tissue repair and regeneration, multiple cell types, hormones, and cell microenvironments must be considered. This is where TE models using biomaterials engineered to mimic native tissue are critical.

The second major challenge is reproducibility given the large number of variables involved in the process including age of cells, hormone levels, socioeconomic background, ethnicity, and other genetic and epigenetic factors. For example, while 17*β*-estradiol is often studied as it is the main circulating estrogen, additional sex hormones such as progesterone, relaxin, and testosterone play a role in tissue repair, regeneration, and homeostasis and naturally occur from sub-nano to micromolar concentrations *in vivo*. In addition, exogenous sex hormones are commonly taken as contraceptives, and HRT to offset symptoms of menopause and aging in males; however, these patients are excluded in clinical trials. Technological advancements must be used to methodically determine the driving parameters that dictate sex differences in tissue repair and regeneration in models that mimic native tissue to generate clinically relevant data.

### Advances in science and technology to meet challenges

Excitingly, the field is at the cusp of addressing the challenges in determining sex differences in tissue repair and regeneration. In our opinion, there are four major advancements that are critical to achieve this endeavor: the -omics revolution, physiologically relevant tissue engineered models, machine learning/artificial intelligence, and high throughput *in vitro* models.

**Figure 16.**
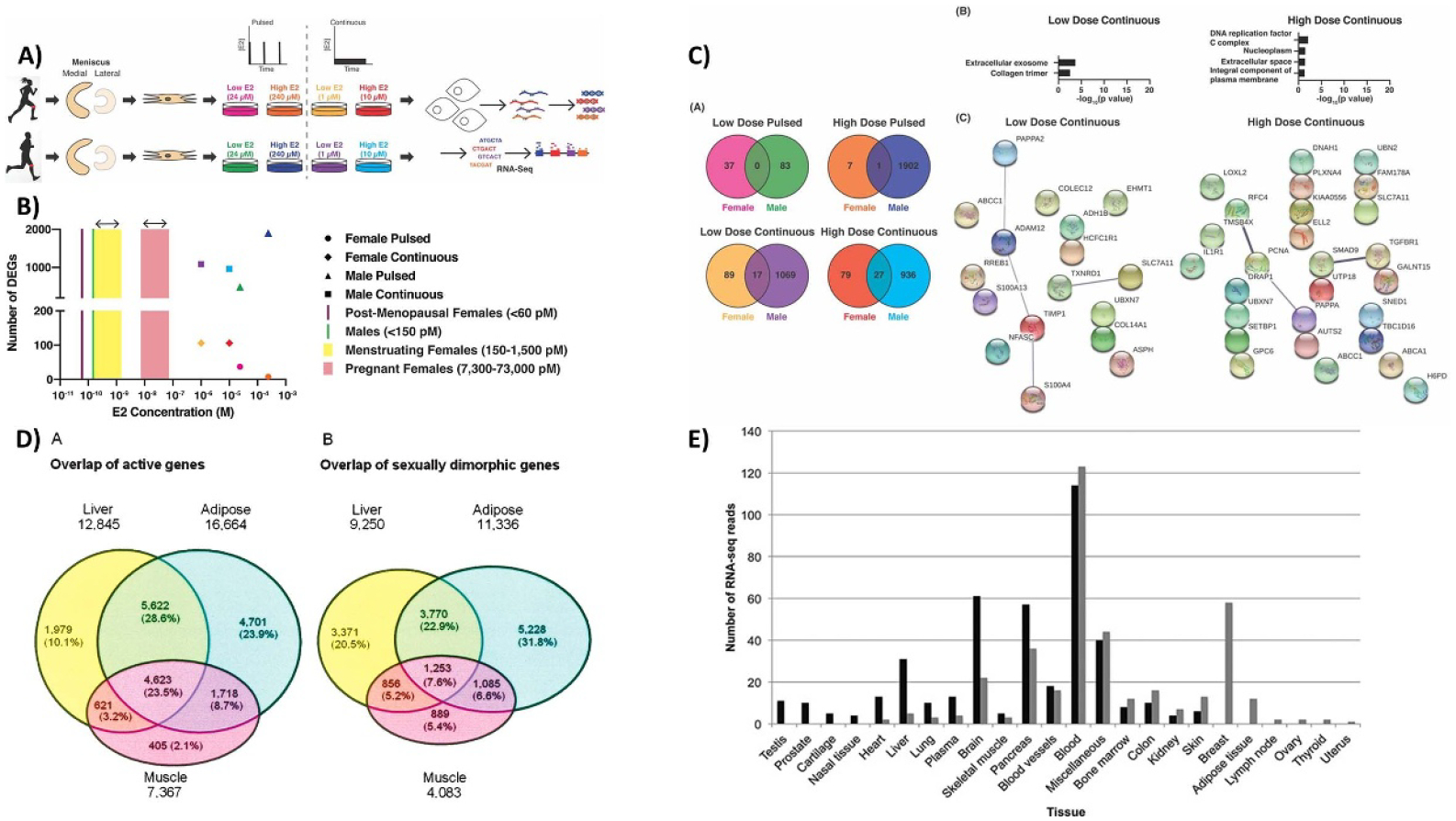
Cells were isolated from the medical meniscus of two 47 year old donors, one of each sex, and treated with two concentrations of E2 under pulsed and continuous conditions. RNA-Seq was then used to analyze the samples. (A) Visualization of the number of differentially expressed genes (DEGs) by treatment group in comparison to physiological serum estrogen concentrations. (B) Analysis of the response of both sexes to the same treatment. Venn diagrams (Venny 2.1) were generated to show which genes were altered in both sexes under the same treatment. For treatments with more than one common gene, functional enrichment analysis was performed using DAVID and STRING analysis to determine what types of genes were differentially expressed. Functional enrichment analysis shows the Gene Ontology Cellular Component groups with *p* values < 0.05. STRING analysis shows known and predicted interactions between the proteins encoded by the genes in confidence views, where associations with stronger data support are thicker. (C) Venn diagram showing tissue specificity of the active genes and the sexually dimorphic genes in murine liver, adipose tissue, and muscle. (D) Reproduced with permission from [174]. Number of male and female human adult RNA-seq reads currently available in NCBI for various tissues. Bar graph depicting the number of adult male and female RNA-seq reads publicly available through NCBI’s Sequence Read Archive (SRA) database in December 2014. (E) [175] John Wiley & Sons. © 2015 Wiley Periodicals, Inc.

Using transcriptomics, proteomics, metabolomics, lipidomics, etc, at the single cell scale, more holistic, large data sets are generated to better understand differences in male and female samples. Figure 16 highlights the tissue-specific genes that are likely responsible for sex different responses in meniscal fibrochondrocytes dosed with estrogen (figures 16(A)–(C)) [163], in liver and adipose tissue [174–176] (figure 16(D)), and a systemic review of differential gene transcripts over multiple tissue types [175] (figure 16(E)) using transcriptomics. As another example, proteomics using techniques such as tandem mass spectroscopy provides full investigation of changes to the ECM proteins, information used to mimic the chemistry and structure of the native ECM in biomaterial design. Physiologically relevant TE models using biomaterials are needed to fundamentally interrogate the components and pathways driving these sex differences. It has rapidly become apparent that 2D models do not accurately recapitulate the native structural, mechanical, and biochemical microenvironment of native tissues and fail to provide the signaling that cells recognize *in vivo*. Comparing the microenvironment of male and female tissue, there is evidence that collagen fiber structure, orientation, and resulting mechanical properties differ [5, 177–179]. A 3D scaffolds and cellular aggregates provide solutions to study crosstalk between multiple cell types and allow for precise and controlled mechanical testing. Knowledge gained from these *in vitro* models will be imperative for design of biomaterial products that promote sex-specific repair including the ability to control release of sex hormones based on promising pre-clinical data for skin and bone regeneration [179]. TE incorporates recent advances in genetic engineering in CRIPSR/CAS9 and lentiviral vector systems to interrogate and differentially regulate genes of interest as revealed by the transcriptomics. Machine learning and artificial intelligence can be used to determine novel and optimal combinations of biomaterial properties and cell–material interactions for more targeted synthesis and testing with decreased time and cost. Further, microfluidics allows for precise, nano-to-micron scale and high throughput control of a cell’s microenvironment. One example of their usage is in generating concentration gradient devices that allow the investigation of a large range of biologic concentrations with less time and cost.

### Concluding remarks

It is becoming more widely known that biological sex plays a role in tissue repair and regeneration as guided by the immune and stem cells that orchestrate these processes. However, the factors that guide this response and effect are unknown. For advancements to occur, researchers and clinicians must consider sex differences as a variable in diagnosis and treatment and remove pre-conceived assumptions from previous understanding based on studies prior to sex as a variable being required. Information gleaned from the discussed studies are critical for both biological male and female health as sex hormone signaling and cell response to microenvironmental changes is important for physiological development, tissue repair, regeneration, and homeostasis in both sexes. Increasing this understanding is imperative for biomaterial product design to promote sex specific regenerative pathways.

### Acknowledgments

J B and J R acknowledge support from NIH NIGMS R35GM143081 and KU Startup Funds. The content is solely the responsibility of the authors and does not necessarily represent the office views of NIH or KU.

## 12. The effects of hormones and pregnancy on the mechanical properties of bone

*Patricia K Thomas*^1,2,3^ *and Anthony G Lau*^1^

^1^ The College of New Jersey, Department of Biomedical Engineering, Ewing, NJ, United States of America

^2^ Department of Biomedical Engineering, Wake Forest University School of Medicine, Winston-Salem, NC, United States of America

^3^ Virginia Tech-Wake Forest University School of Biomedical Engineering and Sciences, NC, United States of America

### Status

**Figure 17.**
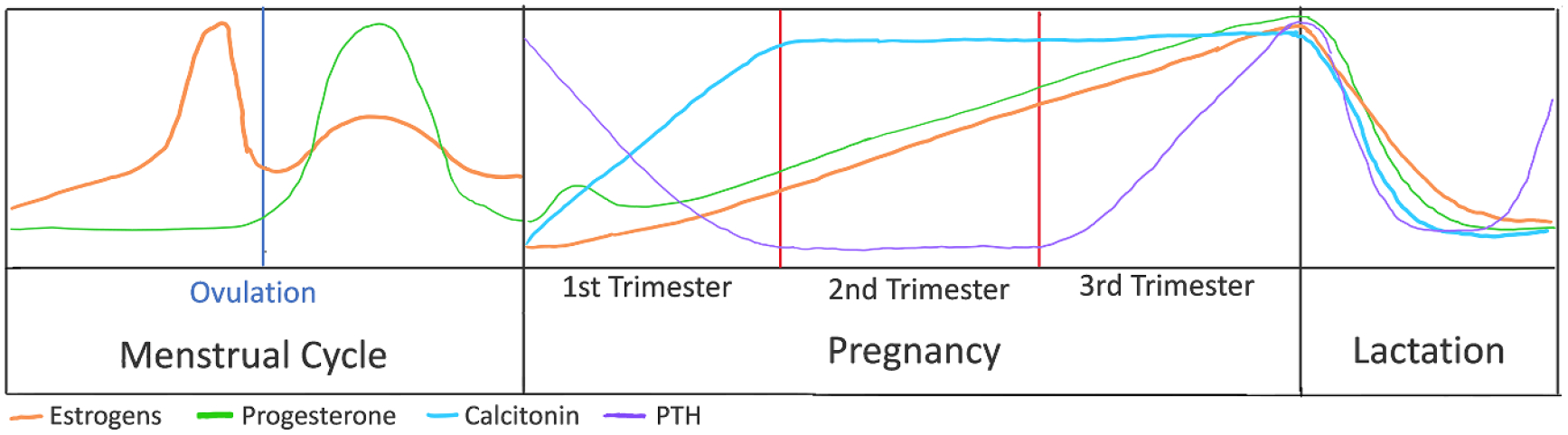
Graph depicting general trends of hormones during portions of the reproductive cycle. *Note*: These are not drawn to scale.

Hormones fluctuate throughout a female’s life cycle. There is a monthly menstrual cycle of hormones that occurs unless pregnancy takes place. Pregnancy has its own cycle of hormones throughout the 40 weeks, followed by a period of lactation. In states of pregnancy, postpartum, and non-pregnancy, there are hormone level changes which include progesterone, estrogens, LH, and FSH (figure 17). These changing hormone levels throughout each cycle affect all tissues of the body, whether it is increasing joint laxity or affecting bone strength [176, 181–183]. For example, the rise in estrogen seen in the menstrual cycle is associated with increased joint laxity in sports medicine [176]. However, these sex-specific hormones are not the only hormones affecting these tissues. Other hormone levels, such as calcitonin and parathyroid hormone (PTH), which both regulate osteoclast activity in bone, have been shown to change over the course of pregnancy [183, 184]. Most female bone health research has been focused on the post-menopausal period of life [185], with limited studies on the bone changes that occur during pregnancy. The bone changes around pregnancy are important to understand, as they set the trajectory of bone health leading up to the onset of menopause.

### Pregnancy

During pregnancy, there are many physiological and hormonal changes that can impact bone health. It is well known that typical pregnancies affect both total calcium and bone metabolism hormone levels. While ionized calcium levels and phosphate levels in the blood are constant throughout the pregnancy in humans, the total serum calcium, which includes ionized, complexed, and albumin bound fractions of calcium, decreases [182, 184, 186–189]. In contrast, total vitamin D levels increase early in pregnancy, and free vitamin D levels are found to increase during the third trimester [182, 184, 186, 188]. The increased vitamin D leads to an increase in calcium absorption in the intestines, allowing for the mother to maintain the ionized calcium levels. Hormonally, calcitonin levels are increased, promoting bone formation [183, 184]. However, it is difficult to determine the state of bone remodeling because serum markers are an indirect measurement of bone remodeling, and there are confounding effects with other hormones.

One study in humans found that urinary cross-linked Type I collagen N-telopeptide (NTx), a biomarker for bone resorption, peaked in the third trimester and postpartum [189]. The study also noted that estrogens also increased throughout pregnancy with further increases after birth. Along with estrogen increase, there was a decrease in total calcium levels in the serum. In humans, bone mineral density (BMD) in the spine and trochanter of the femur was also found to decrease significantly between baseline and postpartum [189]. The hip and femoral neck BMD did not change significantly, likely due to Wolff’s law compensating for the increased weight on these bones.

In pregnant rats, no significant differences in torsional mechanical properties of femora were found until day 20 of a 22 d gestation [182]. However, the pregnant rats had smaller fracture angles, greater stiffness, and lower shear moduli compared to controls. Histologically, there were larger bone crystals at day 20 than controls. This leads to fewer crystals in the same volume, allowing for fractures to propagate more easily, even though the mineral content was not significantly different. There was also an increase in vasculature in the cortical bone of pregnant samples, which would also affect the material properties that were not directly tested [182].

### Postpartum and lactation

Postpartum, bone appears to temporarily demineralize to meet calcium requirements during lactation. This is suspected to be caused by low estrogen levels but might also be caused by PTH-related protein (PTH-rP), which increases during pregnancy and stays elevated during lactation [183, 184]. The mean ionized calcium level appears to increase along with serum phosphorous levels, but PTH, free and bound vitamin D levels fall after birth [184]. Calcitonin increases at the beginning of lactation, likely to either counteract or cause the increased calcium and phosphorous levels.

This increased bone turnover results in losses of BMD in animal models and humans. In humans, most of the mineral loss is in the trabecular bone, where bone density falls 3%–10%, with smaller losses in the cortical bone. This is compared to women of reproductive age with low estrogen levels who have 1%–4% losses in trabecular bone density and none in cortical bone [190, 191]. While estrogen likely contributes to the decreased bone density, it is possible that PTH-rP is also contributing to the accelerated loss of BMD. Similarly, to humans with low estrogen levels, rats with low estrogen levels did not experience as much bone loss as the pregnant rats [183].

During lactation, but not directly after birth, the maximum load on lumbar vertebrae and the vertebral flexural rigidity decreases 64% and 56% respectively, in rats. This is followed by a recovery at about 8 weeks after weaning [183]. In contrast, the maximum load of the femur was not different at birth but was 26% lower at weaning. This could be due to the loss of the weight from the birth of the offspring, causing bone resorption. At 8 weeks recovery after weaning, the max load on the femur had increased compared to at weaning but was still 16% lower than controls. However, geometrically, the average cross-sectional area of the cortical bone decreased during pregnancy and did not recover at 8 weeks after weaning. Overall bone strength was compromised during both pregnancy and lactation but showed recovery after weaning. This suggests that the material properties were compromised during this period and were recovered, while the structural properties of bone did not recover. It is important to note the integrity of both the bone material and structure are important for maintaining overall bone health and strength. If either one remains deficient after pregnancy, there could be long-term bone health risks.

Despite the potential for long term bone health risks after pregnancy, there is some evidence on the contrary. One study found pregnancy reduced risk of hip and vertebral fractures in post-menopausal women, with each additional pregnancy reducing the risk of hip fracture by 9%. The women who had children had the same BMD as women who did not have children, making the diminished risk likely due to geometrical changes in the bone structure [192].

### Current and future challenges

Existing studies show holes in knowledge focusing on material properties of tissue and how they change throughout and after pregnancy. There are also gaps with regards to how hormones and specific pathways affect the bone tissue during and after pregnancy. While there is a better understanding of the short-term effects on both material and mechanical properties and fracture risk than on the long-term effects, there is a lack of understanding for the effects of one singleton pregnancy compared to multiparity pregnancies or multiple pregnancies. One major challenge for addressing these knowledge gaps is the lack of tools to perform robust longitudinal assessments of bone health beyond measures of bone density, especially without exposing the subject to significant doses of ionizing radiation, which is known to be detrimental to bone health.

### Advances in science and technology to meet challenges

Recent developments in peripheral quantitative computed tomography (pQCT) systems are yielding higher image resolution, while lowering radiation doses, and are becoming more common for clinical/research use in humans. However, how these lower dose radiation exposures affect both short- and long-term bone health in humans is still not well understood. There have also been developments for using high resolution MRI to quantify bone microarchitecture, however, there are still some limitations for image resolution at the available magnetic field strengths and the length of time for acquiring these scans.

### Concluding remarks

The microstructural, geometric, and material property changes all need to be evaluated during specific periods of an individual’s life, particularly during the reproductive cycle (i.e. menstruation, pregnancy, lactation, menopause), where there are significant hormonal changes. An understanding of how the hormonal changes affect the components that contribute to overall bone strength can be used to evaluate injury risks as well as novel biomaterials for bone in women.

## Figures and Tables

**Figure 3.**
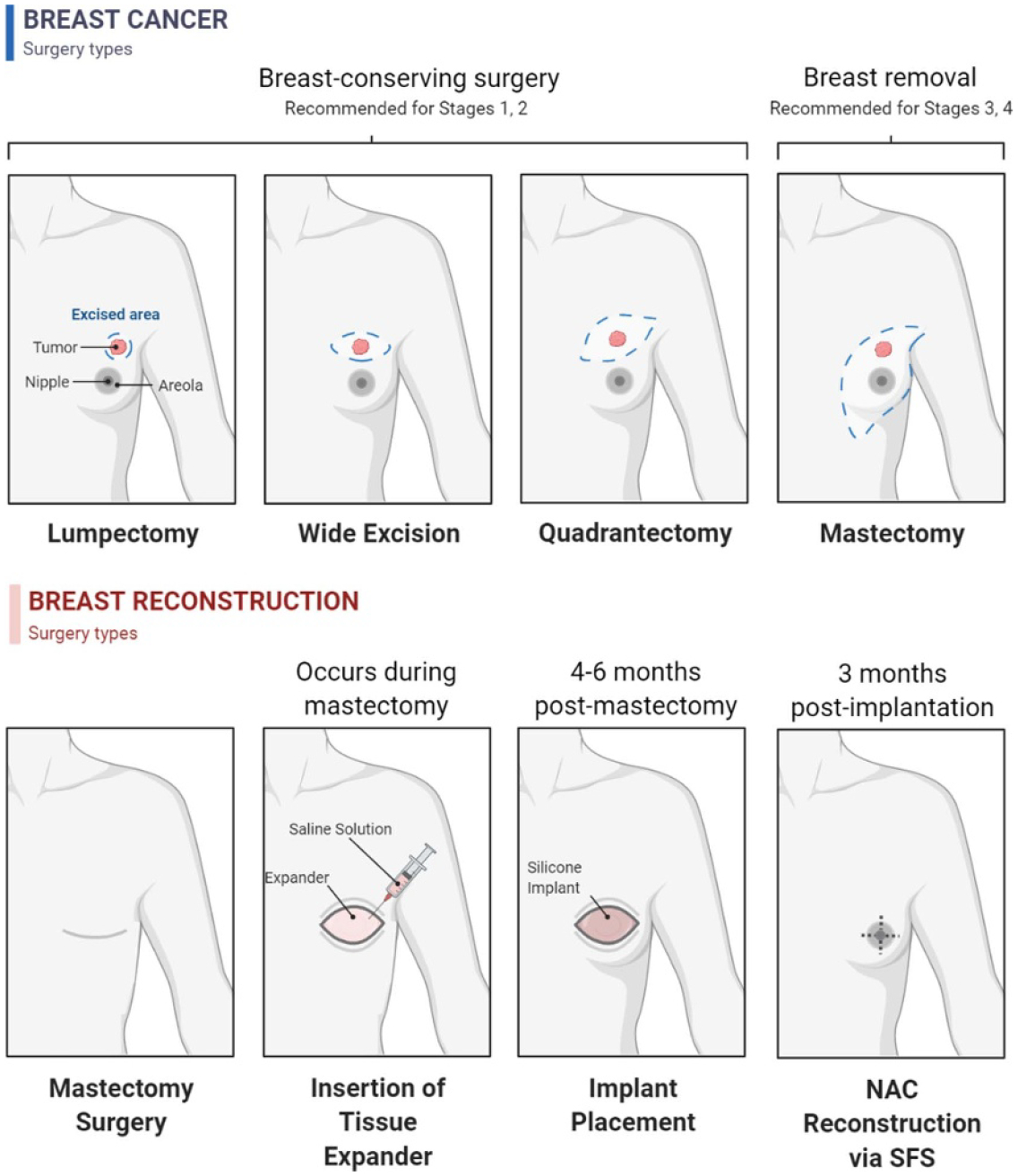
Schematic of current breast cancer surgical and reconstructive techniques. While BCS is preferred, later stage cancers require breast removal, which can be supplemented with reconstruction. Created with BioRender.com.

**Figure 4.**
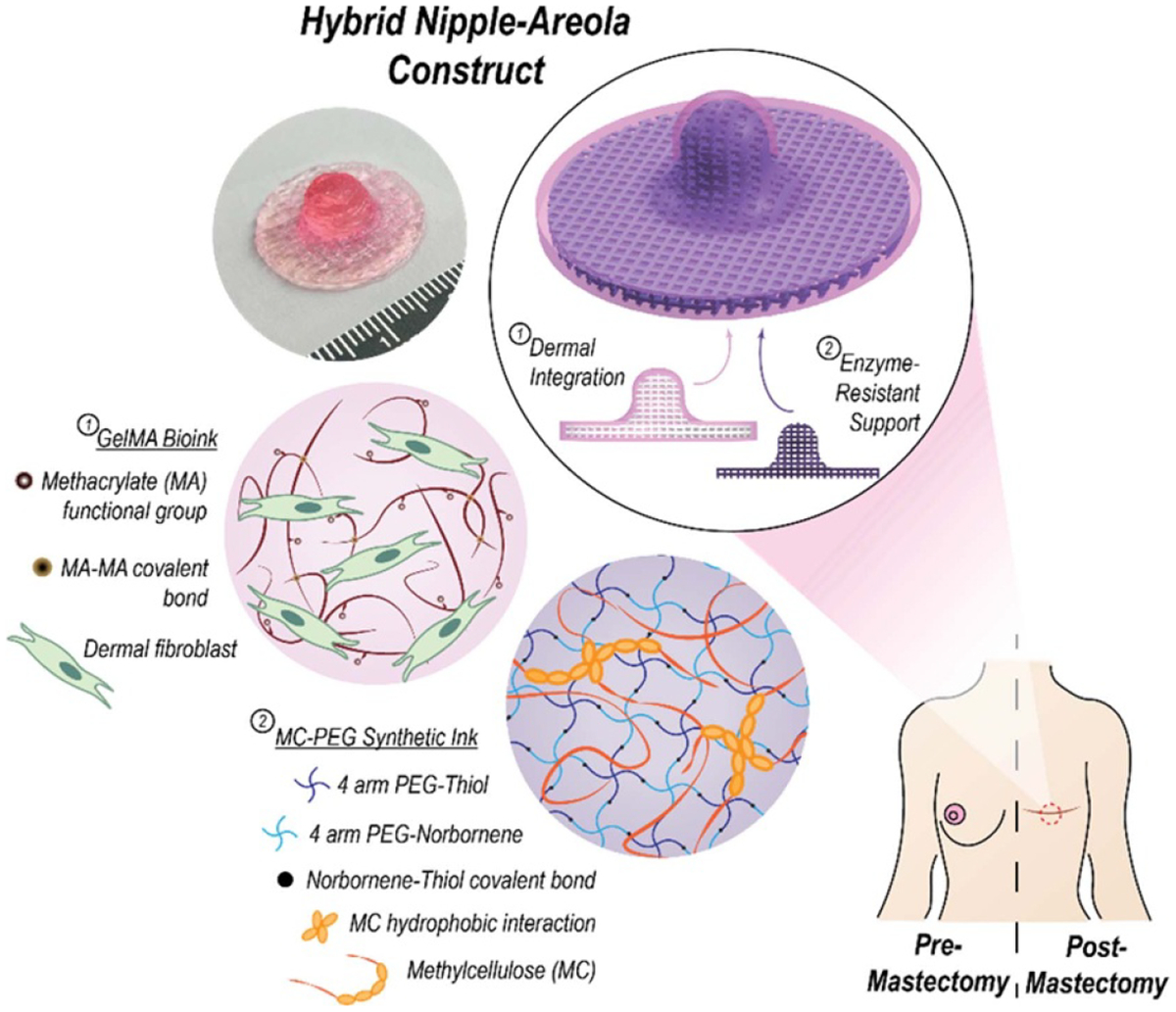
Components and design of GelMA and MC-PEG based bioprinted NAC scaffold. MC-PEG provides structural support in patterned scaffolds, while GelMA serves as a biodegradable ink to influence host tissue integration. [19] John Wiley & Sons. © 2021 Wiley-VCH GmbH.

## Data Availability

The data that support the findings of this study are available upon reasonable request from the authors.
